# Analogous Metabolic Decoupling in Pseudomonas putida and Comamonas testosteroni Implies Energetic Bypass to Facilitate Gluconeogenic Growth

**DOI:** 10.1128/mbio.03259-21

**Published:** 2021-12-14

**Authors:** Rebecca A. Wilkes, Jacob Waldbauer, Ludmilla Aristilde

**Affiliations:** a Department of Biological and Environmental Engineering, Cornell University, Ithaca, New York, USA; b Department of Civil and Environmental Engineering, Northwestern University, Evanston, Illinois, USA; c Department of the Geophysical Sciences, University of Chicago, Chicago, Illinois, USA; University of Oklahoma; University of Hawaii at Manoa

**Keywords:** metabolomics, gluconeogenesis, bacteria, *Pseudomonas*, *Comamonas*, proteobacteria

## Abstract

Gluconeogenic carbon metabolism is not well understood, especially within the context of flux partitioning between energy generation and biomass production, despite the importance of gluconeogenic carbon substrates in natural and engineered carbon processing. Here, using multiple omics approaches, we elucidate the metabolic mechanisms that facilitate gluconeogenic fast-growth phenotypes in Pseudomonas putida and Comamonas testosteroni, two *Proteobacteria* species with distinct metabolic networks. In contrast to the genetic constraint of *C. testosteroni*, which lacks the enzymes required for both sugar uptake and a complete oxidative pentose phosphate (PP) pathway, sugar metabolism in P. putida is known to generate surplus NADPH by relying on the oxidative PP pathway within its characteristic cyclic connection between the Entner-Doudoroff (ED) and Embden-Meyerhoff-Parnas (EMP) pathways. Remarkably, similar to the genome-based metabolic decoupling in *C. testosteroni*, our ^13^C-fluxomics reveals an inactive oxidative PP pathway and disconnected EMP and ED pathways in P. putida during gluconeogenic feeding, thus requiring transhydrogenase reactions to supply NADPH for anabolism in both species by leveraging the high tricarboxylic acid cycle flux during gluconeogenic growth. Furthermore, metabolomics and proteomics analyses of both species during gluconeogenic feeding, relative to glycolytic feeding, demonstrate a 5-fold depletion in phosphorylated metabolites and the absence of or up to a 17-fold decrease in proteins of the PP and ED pathways. Such metabolic remodeling, which is reportedly lacking in Escherichia coli exhibiting a gluconeogenic slow-growth phenotype, may serve to minimize futile carbon cycling while favoring the gluconeogenic metabolic regime in relevant proteobacterial species.

## INTRODUCTION

**FIG 1:**
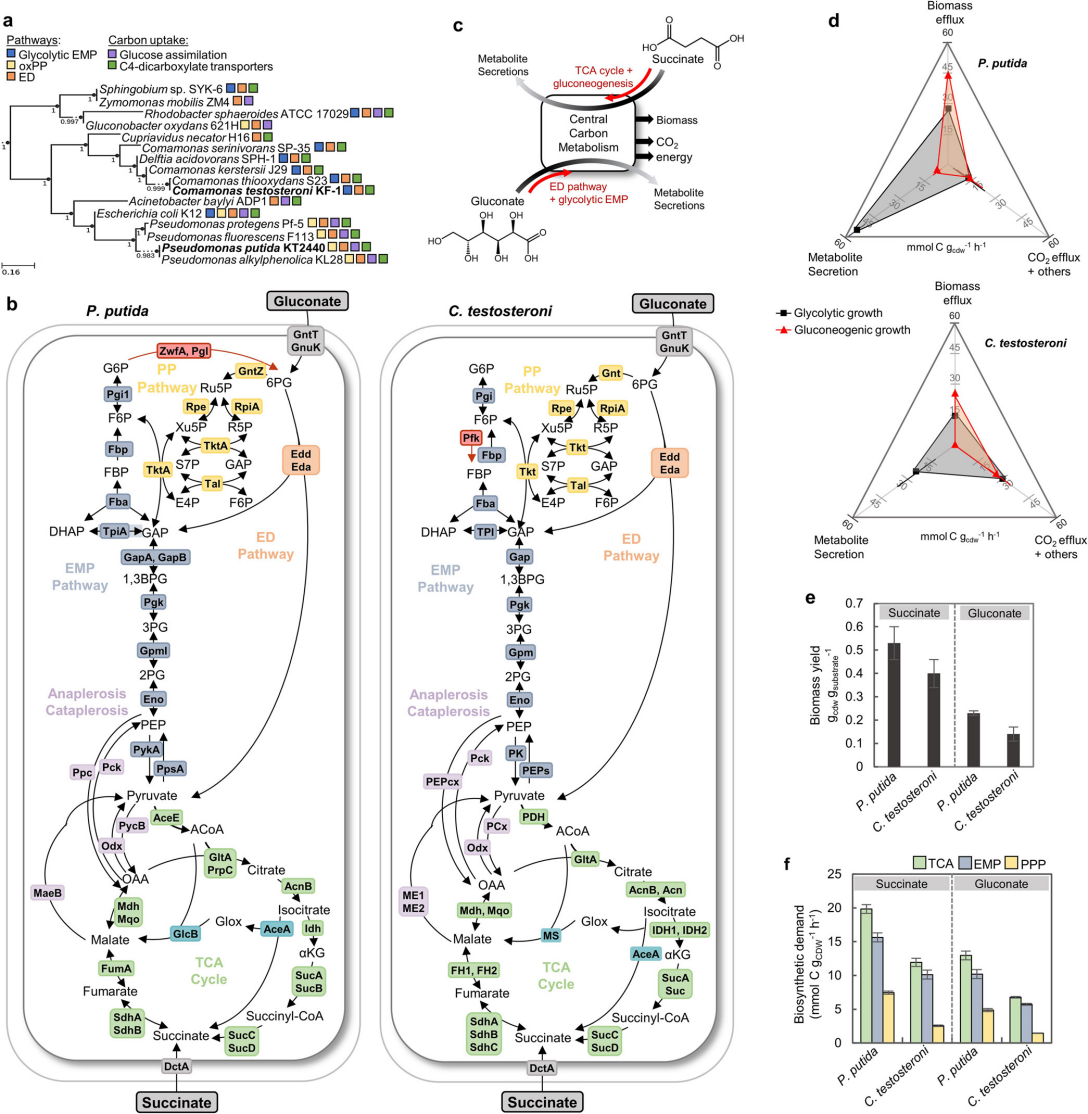
Metabolic pathways for carbon utilization and biomass production. (a) Phylogenetic tree of biotechnologically important bacteria in the phylum *Proteobacteria* constructed using KBase (55). Species in bold represent the organisms studied in this paper. Functional metabolic pathway characteristics (glycolytic EMP pathway, oxidative PP pathway, ED pathway, glucose catabolism, and C4-dicarboxylate uptake) were determined from KEGG genome analyses and metabolism studies (Table S1). Support values are indicated at each branch point and were determined from 1,000 bootstrap replicates (56). (b) Overview of schematic of metabolic pathways and enzymes in P. putida KT2440 and *C. testosteroni* KF-1—initial succinate and gluconate catabolism (gray), EMP pathway (dark blue), ED pathway (orange), PP pathway (yellow), TCA cycle (green), glyoxylate shunt (turquoise), and anaplerosis/cataplerosis reactions (purple). Enzymes and pathways that are only present in P. putida KT2440 or *C. testosteroni* KF-1 are highlighted in red. The full names of proteins abbreviated and the ORF numbers are listed in Table S2. (c) Schematic representation of the two substrates (gluconate and succinate) used to investigate carbon flux partitioning between glycolytic and gluconeogenic metabolic regimes, respectively. (d) Partitioning of total carbon uptake rates in P. putida KT2440 or *C. testosteroni* KF-1 into biomass efflux rate, metabolite secretion rate, and other efflux rates during growth on succinate or gluconate. (e) Substrate-dependent biomass yield determined for P. putida KT2440 and *C. testosteroni* KF-1 grown on succinate or gluconate. (f) The calculated biomass efflux rate required from the TCA cycle, PP pathway, and EMP pathway to sustain biomass growth was calculated from substrate- and species-specific growth rates combined with cellular stoichiometries and genome-based metabolic pathways for P. putida KT2440 and *C. testosteroni* KF-1. In panels e and f, error bars represent the mean ± standard deviation of three biological replicates. Metabolite abbreviations for panel b are as follows: glucose-6-phosphate, G6P; fructose-6-phosphate, F6P; fructose-1,6-bisphosphate, FBP; dihydroxyacetone phosphate, DHAP; glyceraldehyde-3-phosphate, GAP; 6-phosphogluconate, 6PG; ribulose 5-phosphate, Ru5P; xylulose-5-phosphate, Xu5P; ribose-5-phosphate, R5P; sedoheptulose-7-phosphate, S7P; erythrose 4-phosphate, E4P; 1,3-biphosphoglycerate, 1,3BPG; 3-phosphoglycerate, 3PG; 2-phosphoglycerate, 2PG; phosphoenolpyruvate, PEP; acetyl-CoA, ACoA; oxaloacetate, OAA; α-ketoglutarate, αKG.

The phylum *Proteobacteria* consists of metabolically diverse, Gram-negative bacteria widely acknowledged for their importance in medical, agricultural, environmental, and industrial applications (1). Despite the critical relevance of gluconeogenic carbon metabolism in natural carbon processing and biotechnology, how the underlying metabolic network is structured and regulated in proteobacterial species is not well understood. Previous studies (e.g., Escherichia coli [2, 3], Pseudomonas putida [4, 5], Zymomonas mobilis [6, 7], Rhodobacter sphaeroides [8], and Gluconobacter oxydans [9, 10]) have highlighted the selective usage of the Embden-Meyerhoff-Parnas (EMP), pentose phosphate (PP), and Entner-Doudoroff (ED) pathways for carbon flux and redox balance during the metabolism of glycolytic carbon substrates (Fig. 1a, Table S1). However, much remains unknown about the relevance of these glycolysis-associated pathways to the metabolism of gluconeogenic carbon substrates, which feed directly into the tricarboxylic acid (TCA) cycle (11–15). Notably, the TCA cycle represents a hub for energy production (i.e., substrate-level phosphorylation and NADH/ubiquinol [UQH_2_] production), anabolic precursors (specifically, oxaloacetate [OAA] and α-ketoglutarate [αKG]), and NADPH generation (through isocitrate dehydrogenase). Therefore, fluxes into the TCA cycle from bacterial processing of gluconeogenic substrates require partitioning of carbon flux between catabolism, anabolism, and energy generation (11–15). For E. coli, a well-studied model proteobacterial species, slower growth and lower biomass yield during growth on gluconeogenic substrates relative to glycolytic substrates implied ineffective partitioning of carbon flux during metabolism of gluconeogenic substrates (16–18). Here, we investigated two *Proteobacteria* species which exhibit preference for gluconeogenic substrates over glycolytic substrates (14, 19–21) and are expected to exhibit distinct metabolic regulation or genetic constraints during gluconeogenic carbon metabolism—Pseudomonas putida (*Gammaproteobacteria*) and Comamonas testosteroni (*Betaproteobacteria*) (Fig. 1b, Table S2).

10.1128/mbio.03259-21.1 TABLE S1 Functional traits determined for species shown in phylogenetic tree (see Fig. 1a in the main text). KEGG annotations for the genomes of each species were examined for the genes needed for a functional pathway. *Used the Comamonas thiooxydans CNB-1 genome in the KEGG database. **Used *C. testosteroni* TK102 for KEGG identifiers and MetaCyc to confirm for *C. testosteroni* KF-1. Download Table S1, PDF file, 0.3 MB. Copyright © 2021 Wilkes et al. 2021 Wilkes et al. https://creativecommons.org/licenses/by/4.0/ This content is distributed under the terms of the Creative Commons Attribution 4.0 International license.

10.1128/mbio.03259-21.2 TABLE S2 (A) Fold changes in protein abundances in succinate-grown relative to gluconate-grown P. putida KT2440. Values in bold represent *P* values considered significant after adjusting for the false-discovery rate (*q = *0.05). Data were obtained from four biological replicates. NF, not found; N/A, not applicable. (B) Fold changes in protein abundances in succinate-grown relative to gluconate-grown *C. testosteroni* KF-1. Values in bold represent *P* values considered significant after adjusting for the false-discovery rate (*q = *0.05). Data were obtained from four biological replicates. NF, not found; N/A, not applicable. Download Table S2, PDF file, 0.3 MB. Copyright © 2021 Wilkes et al. 2021 Wilkes et al. https://creativecommons.org/licenses/by/4.0/ This content is distributed under the terms of the Creative Commons Attribution 4.0 International license.

Pseudomonas species are attractive biocatalysts due to their repertoire of catabolic genes for diverse substrates (22, 23). These species, which lack phosphofructokinase in the glycolytic EMP pathway (24), employ the ED pathway for glucose catabolism and a well-established EDEMP pathway that cycles carbon through the gluconeogenic EMP pathway, oxidative PP pathway, and back to the ED pathway (4, 5, 25) (Fig. 1b). During glycolytic carbon metabolism, the activity of glucose 6-phosphate (G6P) dehydrogenase in the oxidative PP pathway is implicated in generating a surplus of NADPH, which is important to the biosynthesis of cell components, the production of specialized metabolites, and the protection against oxidative stress (26–28). However, prior studies of P. putida (29, 30) and P. aeruginosa (31) grown in the presence of gluconeogenic carbon substrates (benzoate and acetate, which promoted high flux through NADPH-generating isocitrate dehydrogenase) demonstrated that gluconeogenic metabolism of these substrates was characterized by high carbon flux in the TCA cycle, minimal to no flux through the oxidative PP pathway or ED pathway, and an NADPH surplus. However, the regulation of the cellular metabolome and the associated cofactors in relation to the dependence of gluconeogenic carbon metabolism on TCA cycle flux retention during growth on a gluconeogenic substrate that does not directly support flux into isocitrate dehydrogenase remains to be determined.

*Comamonas* species, which are found in polluted environments (e.g., wastewater-activated sludge and heavy metal-contaminated soils) utilize gluconeogenic substrates almost exclusively (15, 21, 32). Unlike Pseudomonas and other related *Proteobacteria*, *C. testosteroni* strains do not possess the genes associated with G6P dehydrogenase in the oxidative PP pathway, but they encode functional EMP and ED pathways (Fig. 1a and b) (32, 33). Lacking both transporters and phosphorylation enzymes for carbohydrates, strains of *C. testosteroni* are not capable of carbohydrate assimilation (21, 33) and typically rely on gluconeogenic carbon flux to upstream pathways to support biosynthetic carbon demands; gluconate affords an exception by feeding into upper glycolysis via the PP pathway or into lower glycolysis via the ED pathway (Fig. 1b and c). Recently, *C. testosteroni* strains have gained importance as a potential bioremediation chassis (21, 32–35), but it remains to be elucidated how *C. testosteroni* resolves the necessary reliance on the TCA cycle for NADPH production with respect to the assimilatory flux of gluconeogenic carbon substrates.

Here, we investigated the metabolic phenotypes of P. putida and *C. testosteroni* during growth on succinate and gluconate toward resolving the different catabolic regimes of gluconeogenesis versus glycolysis, respectively (Fig. 1b and c). The metabolism of succinate, which is a TCA cycle intermediate transported into the cell by a C4-dicarboxylate transporter, involves strict gluconeogenesis from the TCA cycle to the EMP pathway and PP pathway to support cell maintenance and growth (Fig. 1b and c). Additionally, due to the initiation of succinate catabolism after isocitrate dehydrogenase, NADPH production from the TCA cycle in cells grown on succinate would be solely generated through the retention of carbon flux in the TCA cycle (Fig. 1b). Gluconate, a glycolytic organic acid typically produced from glucose oxidation in the periplasm of bacteria, can be oxidized first to 2-ketogluconate (2KGlcn) in the bacterial periplasm or be phosphorylated directly to 6-phosphogluconate (6PG) in the cytosol to be fed into either the ED pathway or the PP pathway before being channeled eventually to the TCA cycle (Fig. 1b). Our central hypothesis was that the oxidative PP pathway would not be required for NADPH production, and instead, both species would remodel their metabolic architecture to streamline gluconeogenic carbon metabolism by optimizing carbon and energy fluxes from the TCA cycle. To test our hypothesis, we employed a multiomics approach consisting of metabolomics, proteomics, and ^13^C-fluxomics to achieve mechanistic elucidation of gluconeogenic carbon metabolism in both species. Our data demonstrate how both species do not rely on the oxidative PP pathway but instead employ metabolic regulation and an energetic bypass to leverage high carbon flux in the TCA cycle to supply energy for both catabolism and anabolism during gluconeogenic growth. Due to the absence of a complete oxidative PP pathway as a widespread metabolic trait in *Proteobacteria* with a gluconeogenic carbon preference, our findings afford the formulation of new hypotheses regarding the metabolic architecture in these bacteria.

## RESULTS

### Promotion of biomass yield over metabolite secretion during gluconeogenic substrate feeding.

During growth on media containing carbon-equivalent substrate concentrations (100 mM C), both species exhibited shorter lag phases (by about 12 h) and higher growth rates (by 35% to 43%, *P < *0.001) with succinate as the substrate compared to gluconate as the substrate (Table S3, Fig. S1). However, carbon uptake rates were comparable, both between species grown on the same carbon substrate and between a single species across both substrates (*P > *0.1) (Table S3). This discrepancy between carbon uptake rates and biomass growth was attributed to the large fraction of carbon uptake (57% and 32% of carbon uptake in P. putida and *C. testosteroni*, respectively) secreted as 2KGlcn in gluconate-grown cells compared to the minimal metabolite secretions in succinate-grown cells (only 10.5% and 0.3% of carbon uptake in P. putida and *C. testosteroni*, respectively) (Fig. 1d, Table S3, Fig. S1). The high carbon loss from gluconate catabolism resulted in lower biomass yield in gluconate-grown P. putida (by 57%) and *C. testosteroni* (by 65%) compared to the succinate-grown cells (*P < *0.01) (Fig. 1e). The relative fraction of biosynthetic demand from key metabolic pathways to support the biomass efflux rate was determined using genome-specific anabolism pathways, reported biomass stoichiometric composition, and our measured growth rates (Fig. 1f). The highest biomass efflux (nearly 50%) was from the TCA cycle (Fig. 1d), indicating that feeding of a gluconeogenic carbon substrate into the TCA cycle would be advantageous to channeling carbons directly to biosynthetic precursors. Collectively, the physiological findings implied substrate-specific metabolome remodeling for carbon and energy metabolism in both species.

10.1128/mbio.03259-21.3 TABLE S3 Physiological characteristics of P. putida KT2440, *C. testosteroni* KF-1, and *C. testosteroni* T-2 grown on succinate or gluconate. NF indicates that the metabolites were not quantified in the extracellular medium. Data are expressed as mean ± the standard deviation of three biological replicates. *Biomass yield accounted for 2-ketogluconate as a substrate in addition to gluconate.**Data in parentheses represent the consumption rate for 2-ketogluconate. Download Table S3, PDF file, 0.1 MB. Copyright © 2021 Wilkes et al. 2021 Wilkes et al. https://creativecommons.org/licenses/by/4.0/ This content is distributed under the terms of the Creative Commons Attribution 4.0 International license.

10.1128/mbio.03259-21.4 FIG S1 Kinetics of metabolites in extracellular medium during growth of P. putida KT2440, *C. testosteroni* KF-1, and *C. testosteroni* T-2 on succinate or gluconate. (A) Kinetics of succinate depletion (green), gluconate depletion (orange), and 2-ketogluconate production (blue) throughout the cellular growth (gray). Data are expressed as the mean ± the standard deviation of three biological replicates. The darker gray area contained within the dashed lines indicates points during exponential growth used to calculate rates. (B) Percent composition of gluconate (orange) and 2-ketogluconate (blue) during exponential growth shown as the average of the three biological replicates. Download FIG S1, TIF file, 1.0 MB. Copyright © 2021 Wilkes et al. 2021 Wilkes et al. https://creativecommons.org/licenses/by/4.0/ This content is distributed under the terms of the Creative Commons Attribution 4.0 International license.

### Depleted metabolite pools and low energy charge indicate carbon investment into biomass generation during gluconeogenic carbon metabolism.

**FIG 2:**
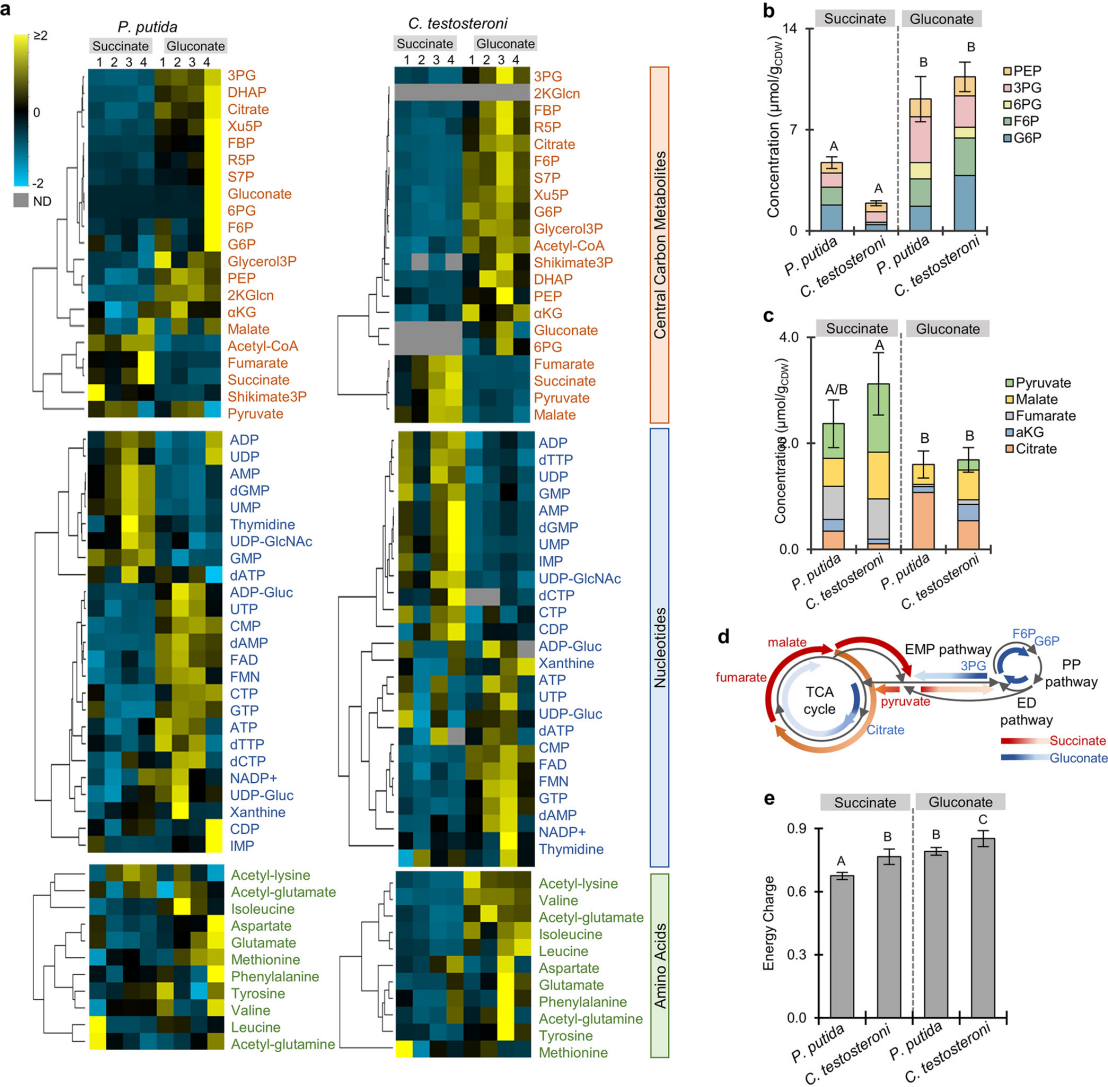
Global metabolome remodeling during growth on a gluconeogenic substrate (succinate) versus a glycolytic substrate (gluconate). (a) Unsupervised hierarchical clustering for each species (P. putida KT2440 or *C. testosteroni* KF-1, four biological replicates denoted as 1 through 4) conducted across intracellular metabolite pools divided into central carbon metabolites, nucleotides, and amino acids. Relative metabolite concentrations are normalized to have a mean equal to 0 and a standard deviation equal to 1. (b) Intracellular pool (μmol/g) of phosphorylated intermediate pools of PEP, 3PG, 6PG, F6P, and G6P. Data are expressed as the mean ± the cumulative standard deviation of four biological replicates. (c) Intracellular pool (μmol/g) of organic acids—pyruvate, malate, fumarate, αKG, and citrate. Data are expressed as the mean ± the cumulative standard deviation of four biological replicates. (d) Schematic summary of proposed metabolic routing of substrate carbons based on relative metabolite pools in in succinate-grown cells (red) versus gluconate-grown cells (blue). (e) Energy charge calculated from the pools of ATP, ADP, and AMP. Data are expressed as the mean ± the cumulative standard deviation of four biological replicates. For panels b, c, and e, statistically significant differences (*P* value less than 0.05) are denoted by a change in letter. The significance was determined using one-way ANOVA followed by Tukey HSD *post hoc* tests. Abbreviations for central carbon the metabolites are as described in Fig. 1.

Of the 69 intracellular metabolites profiled, the abundances of up to 66% of central carbon metabolites, 64% of nucleotides, and 45% of amino acids were elevated consistently across biological replicates of gluconate-grown cells relative to succinate-grown cells (Fig. 2a and Fig. S2). Higher concentrations (by up to 5.5-fold, *P < *0.0004) of phosphorylated intermediates (phosphoenolpyruvate [PEP], 3PG, 6PG, fructose 6-phosphate [F6P], and G6P) during growth on gluconate compared to growth on succinate indicated high carbon accumulation in the ED and PP pathways from gluconate catabolism in both species (Fig. 2b). In contrast, during growth on succinate compared to growth on gluconate, the cumulative pools of TCA cycle-related organic acids were greater by 85% in *C. testosteroni* (*P ≤ *0.005) and by nearly 50% in P. putida (*P = *0.25), although this difference was not statistically significant for P. putida (Fig. 2c). Consistent with the incorporation of succinate into the reductive side of the TCA cycle (Fig. 1b), up to 52% of the cumulative organic acid pools consisted of fumarate and malate (both of which are immediately downstream of succinate), whereas citrate and αKG (from the oxidative side of the TCA cycle) accounted for only 6% of the total organic acid pools in succinate-grown cells (Fig. 2a and c). In sum, the relative metabolite pools across the two different growth conditions highlighted carbon accumulation in selective pathways that were in accordance with the substrate-dependent metabolic regime, promoting carbon availability in the TCA cycle in succinate-grown cells versus the accumulating carbon in the upper EMP pathway and limiting carbon availability in the TCA cycle in gluconate-grown cells (Fig. 2d). Notably, we found that the nucleoside triphosphates were depleted in both species (6 out of 7 in P. putida and 4 out of 7 in *C. testosteroni*) during growth on succinate compared to gluconate (Fig. 2A). Moreover, the energy charge (calculated from the quantified pools of ATP, ADP, and AMP) was up to 18% higher in gluconate-grown cells compared to succinate-grown cells (*P < *0.05) (Fig. 2e); the lowest energy charge recorded for succinate-grown cells of P. putida (0.68 ± 0.02) was still within the minimum requirement reported for healthy cells of E. coli (Fig. 2e) (36, 37). Therefore, the high biomass demand during succinate growth appeared to be a sink for both succinate-derived carbons and the ATP pool, thereby decreasing the carbon available for metabolite secretions or futile carbon cycling through the EMP and PP pathways.

10.1128/mbio.03259-21.5 FIG S2 Global metabolome remodeling of P. putida KT2440, *C. testosteroni* KF-1, and *C. testosteroni* T-2 during growth on gluconeogenic substrate (succinate) versus glycolytic substrate (gluconate). For each species, metabolite pools were extracted for four biological replicates denoted 1 through 4. Unsupervised hierarchical clustering was conducted across the entire species’ metabolome and across intracellular metabolite pools divided into central carbon metabolites, nucleotides, and amino acids. Relative metabolite concentrations are normalized to have a mean equal to 0 and a standard deviation equal to 1. Download FIG S2, TIF file, 0.9 MB. Copyright © 2021 Wilkes et al. 2021 Wilkes et al. https://creativecommons.org/licenses/by/4.0/ This content is distributed under the terms of the Creative Commons Attribution 4.0 International license.

### Curtailed protein production highlights pathways unnecessary for gluconeogenic carbon metabolism.

**FIG 3:**
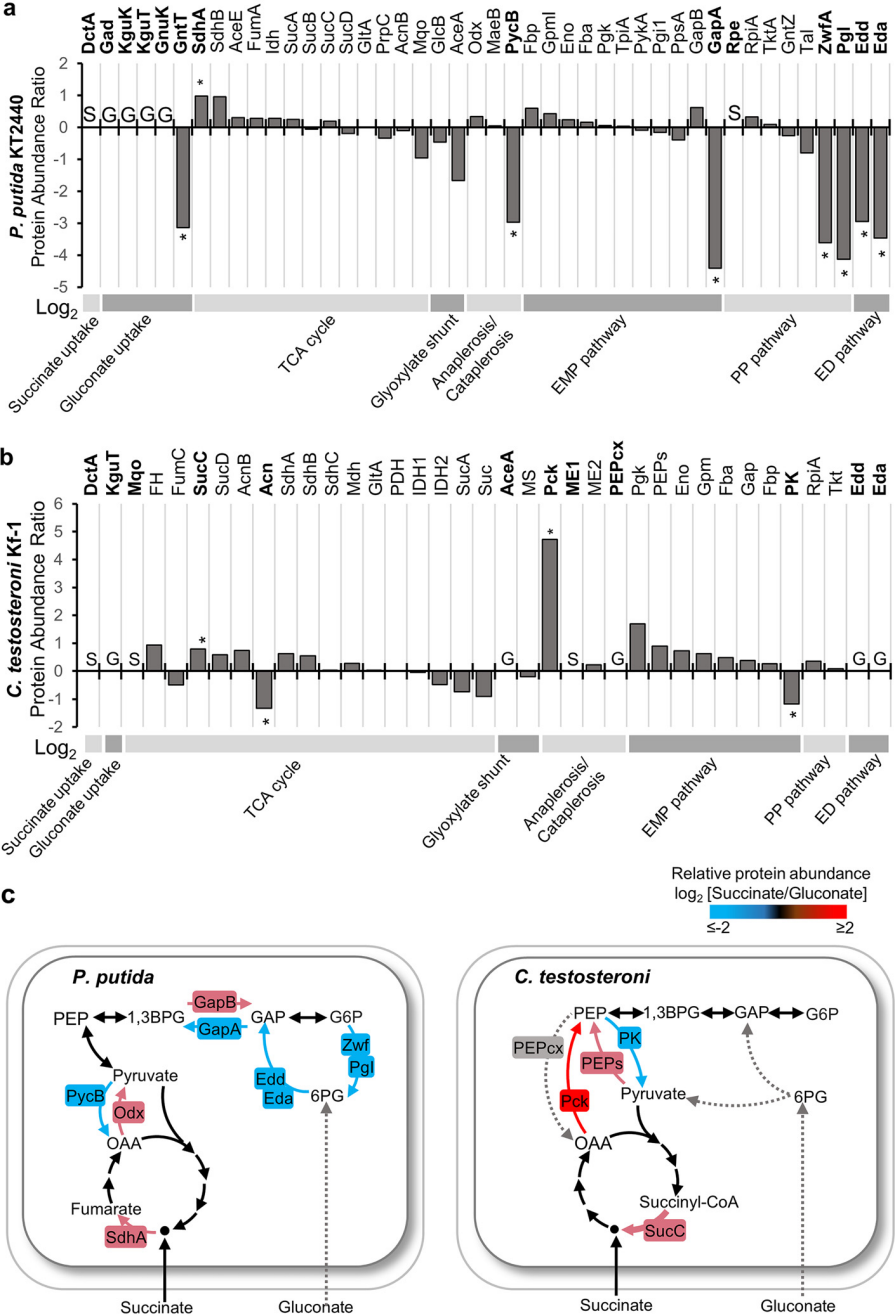
Enzyme-level regulation of selective metabolic nodes to facilitate gluconeogenic carbon flow. (a and b) Shown are the log_2_ fold change in protein content in succinate-grown cells relative to gluconate-grown cells of (a) P. putida KT2440 and (b) *C. testosteroni* KF-1. Protein names in bold represent proteins only found in one growth condition or proteins with significant differences between conditions. The asterisk (*) denotes significant differences in protein abundance ratios with a *P* value less than 0.05 after correction for false-discovery rate. Abbreviations: S, proteins only detected during growth on succinate; G, proteins only detected during growth on gluconate. Data were obtained from four biological replicates. (c) Key species-specific regulation points associated with gluconeogenic growth identified by elevation (shades of red) and depletion (shades of blue) of specific proteins during growth on succinate relative to growth on gluconate. Dotted gray lines indicate that the enzymes in the pathway were only detected during growth on gluconate. Abbreviations for the metabolites are described in Fig. 1.

Transporters and enzymes involved in uptake and initial catabolism of each substrate were only detected during growth on the specific substrate, except for the gluconate transporter GntT in P. putida, but its abundance was 8.5-fold less during growth on succinate compared to growth on gluconate (Fig. 3a and b). Of the 38 proteins quantified for P. putida and 36 proteins for *C. testosteroni* that were associated with central carbon metabolism, nearly 20% were significantly depleted (by up to 21-fold) in succinate-grown cells relative to gluconate-grown cells of both species (Fig. 3a and b, Table S2A and B). All the enzymes in the ED pathway for both species and the oxidative PP pathway for P. putida were absent or depleted (up to 17-fold) during growth on succinate relative to gluconate, revealing minimal involvement of these two pathways during succinate metabolism (Fig. 3a and b). Markedly, we found species-specific regulation of protein abundance in the EMP pathway, whereby control points of glycolytic versus gluconeogenic flux occurred at glyceraldehyde 3-phosphate (GAP) in P. putida and at PEP in *C. testosteroni* (Fig. 3c). Specifically, while there was no significant change in the one quantifiable GAP dehydrogenase enzyme (Gap) in *C. testosteroni*, there was a 21-fold depletion in GapA and a 1.5-fold increase in GapB in P. putida (Fig. 3a to c). This functional partitioning of different Gap enzymes between gluconeogenesis and glycolysis in P. putida was also reported in Bacillus subtilis (16). Furthermore, in *C. testosteroni* but not P. putida, there was a 2.2-fold depletion in pyruvate kinase (PK), whereas there was a near 2-fold elevation in PEP dehydrogenase (PEPs) during growth on succinate relative to growth on gluconate (Fig. 3a to c).

Of the proteins associated with the TCA cycle, only succinate dehydrogenase (SdhA) was significantly elevated (2-fold) in P. putida, and only succinyl-coenzyme A synthase (SucC) was significantly elevated (1.7-fold) in *C. testosteroni* during growth on succinate relative to growth on gluconate (Fig. 3a to c). Additional species-specific regulatory points to control carbon fluxes into or out of the TCA cycle were identified through the relative abundance of anaplerotic and cataplerotic enzymes (Fig. 3a to c). In P. putida, there was an 8-fold depletion of pyruvate carboxylase (PycB; an anaplerotic enzyme) accompanied by a 1.2-fold increase of OAA decarboxylase (Odx; a cataplerotic enzyme) in succinate-grown cells relative to gluconate-grown cells (Fig. 3a and c). In *C. testosteroni*, there was a 26-fold increase in PEP carboxykinase (Pck; a cataplerotic enzyme) during growth on succinate relative to gluconate, whereas PEP carboxylase (PEPcx; an anaplerotic enzyme) was not detected (Fig. 3b and c). Therefore, the protein-level metabolic regulation in P. putida cells was through decreasing the relative abundance of the anaplerotic enzyme at the node between pyruvate and OAA, whereas *C. testosteroni* increased the relative abundance of the cataplerotic enzyme at the node between PEP and OAA (Fig. 3c). In sum, the proteomics data highlighted regulation of gluconeogenic carbon flux by altering the relative abundance of specific proteins toward increasing flux to biomass production while reducing carbon flux to unnecessary pathways (Fig. 3c).

### Metabolic endpoint at G6P highlights disconnection of gluconeogenic carbon metabolism from the oxidative PP pathway and the dependence of both species on the TCA cycle.

**FIG 4:**
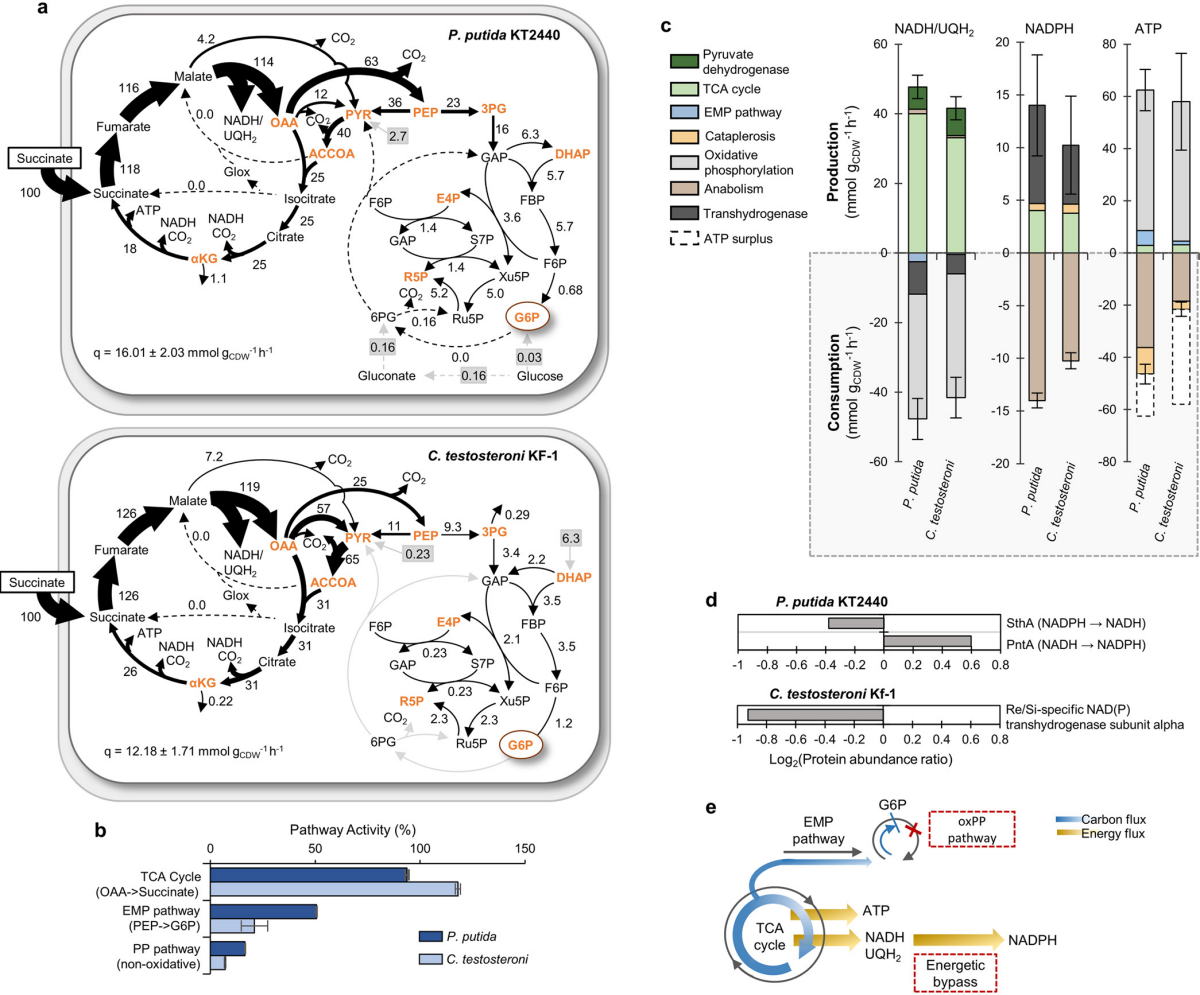
^13^C-metabolic flux analysis of carbon and energy flux partitioning in the gluconeogenic metabolic regime. (a) Metabolic fluxes (expressed in percentage relative to succinate uptake rate [*q*]) in succinate-grown P. putida (top) and *C. testosteroni* (bottom). Metabolites highlighted in orange represent biomass precursors. Gray boxes and arrows represent unlabeled carbon influx from the extracellular matrix. Abbreviations for the metabolites in panel a are described in Fig. 1. (b) Key metabolic pathway activities, shown as percentage (%) of succinate uptake, in P. putida KT2440 (dark blue) and *C. testosteroni* KF-1 (light blue). (c) Absolute production and consumption rates (mmol g_CDW_^−1^ h^−1^) of NADH/UQH_2_, NADPH, and ATP determined from the cellular fluxes and species-specific biomass stoichiometry. Transhydrogenase reactions of NADH to NADPH were invoked to supply the additional NADPH needed for anabolism. ATP production from NADH/UQH_2_ was calculated using a phosphate to oxygen (P/O) ratio of 1.5. Error bars represent the cumulative standard deviation across all pathways contributing to either the production or consumption rate. A breakdown of individual reactions error can be found in Data Set S1. (d) Protein abundances of transhydrogenase enzymes as a log_2_ ratio of succinate-grown cells to gluconate-grown cells. (e) Schematic overview of gluconeogenic carbon fluxes (in blue), including the shutdown of the oxidative pentose phosphate (oxPP) pathway in both species, and the resulting energy fluxes (in dark yellow), including the energetic bypass such as through transhydrogenase reactions, to favor the fast-growth gluconeogenic growth phenotype.

Using ^13^C-tracer carbon mapping and metabolic flux analysis, we determined high fluxes (greater than 100% of uptake) from succinate to OAA within the TCA cycle for both species, consistent with the metabolite pools (Fig. 4a and 2b). The glyoxylate shunt also remained inactive for both species, which would support greater reducing power generation from flux through the oxidative TCA cycle (Fig. 4a). In P. putida, we found that G6P served as the metabolic endpoint whereby the gluconeogenic EMP pathway flux was decoupled from the ED pathway (Fig. 4a), in agreement with the absence of the 6PG pool and the depletion of oxidative PP and ED pathway enzymes in succinate-grown cells relative to gluconate-grown cells (Fig. 2a and 3c). Thus, the gluconeogenic flux (23% of succinate uptake) from PEP to the rest of the EMP pathway in P. putida was sufficient to support biosynthetic demand without necessitating surplus carbon flux through the oxidative PP pathway (Fig. 4a and Data Set S1). Likewise, in *C. testosteroni*, which lacks the genes for the oxidative PP pathway, the gluconeogenic flux (9.3% of succinate uptake) from PEP to the rest of the EMP pathway was sufficient to supply biosynthetic precursors in the EMP and nonoxidative PP pathways (Fig. 4a and Data Set S1).

10.1128/mbio.03259-21.9 DATA SET S1 ^13^C-metabolic flux analysis results and cofactor calculations for P. putida KT2440, *C. testosteroni* KF-1, and *C. testosteroni* T-2. All fluxes were modeled using parallel optimization on OpenFlux2. LB95 and UB95 represent the lower bound and upper bound, respectively, of the 95% confidence intervals obtained using nonlinear sensitivity analysis on OpenFlux2. SD represents the standard deviation of the flux calculated from the UB95 and LB95. Download Data Set S1, XLSX file, 0.03 MB. Copyright © 2021 Wilkes et al. 2021 Wilkes et al. https://creativecommons.org/licenses/by/4.0/ This content is distributed under the terms of the Creative Commons Attribution 4.0 International license.

Carbon mapping analysis of the isotopologue fractions for both species grown on false-[2,3-^13^C]-succinate without washing cells between transfers revealed nonlabelled fractions in EMP pathway metabolites that could not be attributed to carbon rearrangements (see Text S1 and Fig. S3). Unlabeled medium carry-over was found to be responsible for nonlabelled isotope fractions when cells were transferred without a washing step (Fig. S3 to S5). Modeling of carbon fluxes in unwashed cells to include input nodes from potential medium carry-over showed that less than 7% of succinate uptake was from scavenged unlabeled extraneous carbons in both P. putida and *C. testosteroni* (Fig. 4a). This unlabeled carbon influx populated malate and pyruvate to contribute to TCA cycle flux in P. putida but was incorporated mostly into dihydroxyacetone phosphate (DHAP) to support EMP pathway flux in *C. testosteroni* (this was confirmed in two *C. testosteroni* strains, KF-1 and T-2) (Fig. 4a and Fig. S3 and S5). However, markedly similar pathway activity in washed and unwashed cells confirmed that the peripheral carbon scavenging supported biosynthetic flux within a robust metabolic flux network (Fig. S3). Taken together, the metabolite labeling data and flux analysis revealed that, with the absence of an active oxidative PP pathway in both species, the flux retained in the TCA cycle appeared to be pivotal for both species to generate the energy and reducing power required for biosynthetic demand during gluconeogenic growth (Fig. 4b).

10.1128/mbio.03259-21.6 FIG S3 Isotope fractions indicate scavenging of unlabeled carbons to support metabolic fluxes. (A) Carbon mapping of expected labeling patterns from growth on [2,3]-^13^C-succinate and experimentally determined isotope fractions. Blue filled circles are from carbons cycled through the TCA cycle. The blue arrow indicates the pathway only present in P. putida, and the green arrow indicates that the pathway is only present in *C. testosteroni.* Red arrows indicate potential influx locations from the unlabeled LB glycerol stock. (B) Pathway activities represent the cumulative net fluxes of carbon through each of the pathways determined from metabolic flux analysis. For the net fluxes for the washed cell condition and *C. testosteroni T-2* see Data Set S1. (C) Flux precision scores determined comparing the error in optimized ^13^C-metabolic flux analysis between the washed cell condition and the unwashed cell condition for *C. testosteroni* KF-1. A value greater than 1 indicates greater precision after washing the cells. Red arrows represent influx points for unlabeled carbons. (D) Model optimized biomass efflux of *C. testosteroni* KF-1 cells when directly transferred versus when cells were washed between both transfers. The starting biomass efflux input into the models (gray) was calculated from growth rate and biomass stoichiometry. Error bars are the standard deviation of the mean of three replicates for the calculated biomass efflux and six replicates for the model optimized values. Download FIG S3, TIF file, 1.3 MB. Copyright © 2021 Wilkes et al. 2021 Wilkes et al. https://creativecommons.org/licenses/by/4.0/ This content is distributed under the terms of the Creative Commons Attribution 4.0 International license.

10.1128/mbio.03259-21.8 FIG S5 Labeling profiles of metabolites extracted from P. putida KT2440, *C. testosteroni* KF-1, and *C. testosteroni* T-2 during growth on [1,4-^13^C]-succinate or [2,3-^13^C]-succinate. Error bars are the standard deviation obtained from three biological replicates. Refer to Fig. 1 (main text) for metabolite abbreviations. Download FIG S5, TIF file, 2.6 MB. Copyright © 2021 Wilkes et al. 2021 Wilkes et al. https://creativecommons.org/licenses/by/4.0/ This content is distributed under the terms of the Creative Commons Attribution 4.0 International license.

10.1128/mbio.03259-21.10 TEXT S1 Determination of carbon scavenging. Detailed explanation of nonlabelled fractions in isotope data and experimental determination that nonlabelled fractions were obtained from extracellular matrix during medium transfers. Download Text S1, DOCX file, 0.01 MB. Copyright © 2021 Wilkes et al. 2021 Wilkes et al. https://creativecommons.org/licenses/by/4.0/ This content is distributed under the terms of the Creative Commons Attribution 4.0 International license.

### TCA cycle flux sustains energy yield and NADPH production for cellular anabolism.

The ^13^C-fluxomics analysis of succinate-grown cells combined with species-specific biomass stoichiometry revealed that the overall production rates of NADH/UQH_2_, NADPH, and ATP in P. putida (47.69 ± 3.35 mmol NADH/UQH_2_ cell dry weight in grams [g_CDW_]^−1^ h^−1^; 14.01 ± 4.79 mmol NADPH g_CDW_^−1^ h^−1^; 62.45 ± 5.90 mmol ATP g_CDW_^−1^ h^−1^) were comparable to the corresponding rates in *C. testosteroni* (41.59 ± 3.32 mmol NADH/UQH_2_ g_CDW_^−1^ h^−1^; 10.24 ± 4.61 mmol NADPH g_CDW_^−1^ h^−1^; 62.21 ± 7.01 mmol ATP g_CDW_^−1^ h^−1^) (Fig. 4c). In the absence of the oxidative PP pathway, NADPH production was from isocitrate dehydrogenase in the TCA cycle and malic enzyme during cataplerosis, both of which were insufficient to support the biosynthetic NADPH flux demand in both species (Fig. 4c). Transhydrogenase reactions, which can interconvert reduced cofactors, were thus predicted to compensate for the deficit in NADPH production and to account for 66% and 55% of the NADPH produced by P. putida and *C. testosteroni*, respectively (Fig. 4c). In both species, flux through the TCA cycle was primarily responsible for the production of energy and reducing power—99% of NADH/UQH_2_, 93% of NADPH (including the transhydrogenase reactions to convert TCA cycle-derived NADH to NADPH), and 90% of ATP (including oxidative and substrate-level phosphorylation) (Fig. 4c and Data Set S1).

In accordance with the involvement of transhydrogenases, our proteomics analysis identified two transhydrogenase enzymes in P. putida, which exhibited opposing relative levels in succinate-grown cells compared to gluconate-grown cells; transhydrogenase PntA was elevated by 50%, whereas transhydrogenase SthA was depleted by 30%, implying possible differences in the regulation and directionality of these enzymes in P. putida as previously reported in E. coli (Fig. 4d) (38, 39). In *C. testosteroni*, we identified only one transhydrogenase protein (with 55% identity to PntA in E. coli), which underwent a nearly 2-fold depletion during growth on succinate relative to growth on gluconate (Fig. 4d). Due to the bidirectionality of transhydrogenases (28), the single protein identified in *C. testosteroni* may have a different regulatory mechanism than that found in E. coli. Alternatively, a different unidentified mechanism may be contributing to NADPH production in *C. testosteroni*. For instance, we identified ferredoxin:NADP+ oxidoreductases (FNRs, EC:1.18.1.2) in the proteome of *C. testosteroni* KF-1 that was about 40% more abundant during growth on succinate than during growth on gluconate, but only NfnAB-type FNRs are reported to be involved in NADPH generation from NADH (28). Resolving between different potential mechanisms of NADPH production was beyond the scope of our data. Nevertheless, contrasting the NADPH surplus reported previously for glycolytic growth on glucose for P. putida (40) or gluconeogenic growth on acetate for P. aeruginosa (31), our findings demonstrated that the NADPH deficit resulting from gluconeogenic metabolism during growth of both species on succinate could be overcome by transhydrogenase conversion of NADH to NADPH. Accounting for the transhydrogenase conversion of NADH and assuming a conservative value of 1.5 for the phosphate to oxygen ratio (41), we approximated that 86% of ATP was produced from oxidative phosphorylation and that the total ATP yield was more than sufficient to meet biosynthetic demand (Fig. 4c). We attributed the ATP surplus, which was calculated as the difference between the total ATP produced and ATP consumed by anabolism (Fig. 4c), as the ATP required for cellular maintenance and other cellular reactions that were not accounted for in our model. The 60% greater ATP surplus in *C. testosteroni* compared to P. putida was consistent with the aforementioned 15% greater energy charge found for *C. testosteroni* than P. putida (Fig. 2e and 4c).

## DISCUSSION

Comprehensive systems-level understanding of native metabolism in both well-known and novel species is warranted (23, 42). Specifically, quantitative flux analysis of gluconeogenic substrates, which represent common substrates in environmental matrices (43) and industrial nutrient media (11, 44), is largely lacking. Here, we sought to gain new insights on how both P. putida and *C. testosteroni*, two important proteobacterial species and promising biological chasses for bioproduction and bioremediation (21–23, 34, 45–47), streamline their carbon metabolism during gluconeogenic feeding. Unlike the model proteobacterial species E. coli, which preferentially metabolizes glycolytic substrates over gluconeogenic substrates (16, 48), either regulatory or genetic preference for gluconeogenic substrates has been implicated for both P. putida (14, 19, 20) and *C. testosteroni* (21). We found that both of these species optimized their carbon and energy fluxes to support higher biomass growth during gluconeogenic growth relative to glycolytic growth by (i) coupling direct carbon influx into the TCA cycle with a reduction in metabolite secretions to fulfill high biosynthetic flux demand; (ii) decreasing carbon and protein investments in the EMP, oxidative PP, and ED pathways; (iii) promoting carbon flux retention in the TCA cycle at the expense of the gluconeogenic EMP pathway; and (iv) relying on transhydrogenase conversion of NADH to NADPH to maintain sufficient cofactors for cellular growth (Fig. 4e).

Despite the insufficient production of NADPH from the TCA cycle flux to support anabolism, we demonstrate that gluconeogenic carbon metabolism can bypass the absence of the oxidative PP pathway in *C. testosteroni* or an inactive oxidative PP pathway through decoupled EMP and ED pathways in P. putida by leveraging transhydrogenase conversion of NADH to NADPH to meet cellular biosynthetic demands. Along with transhydrogenase enzymes, the oxidative PP pathway through the cyclic EDEMP pathway was identified previously to be crucial to maintaining redox balance during oxidative stress (27, 49, 50). For instance, both P. putida and E. coli cells, which are capable of metabolic control to boost flux through the oxidative PP pathway, were less susceptible to oxidative stress than cells that lacked this metabolic flexibility (27, 51). Therefore, particularly in the absence of the oxidative PP pathway during growth on a variety of gluconeogenic substrates, alternative metabolic strategies to respond to oxidative stress responses should be explored.

The oxidative PP pathway is widely reported to benefit glycolytic metabolism by generating reducing power (4, 51, 52). However, our findings implied that metabolic investment into enzymes for the oxidative PP pathway would be futile and even costly for gluconeogenic metabolism in relevant *Proteobacteria*. Both E. coli and P. putida have operational oxidative PP pathways, but E. coli preferentially utilizes glycolytic substrates (16), whereas P. putida preferentially utilizes gluconeogenic substrates (14, 20). The reported lack of modulation in protein production and reaction flux in the oxidative PP pathway may afford the quick metabolic switch from gluconeogenic metabolism to glycolytic metabolism observed in E. coli, albeit at the cost of slower growth and lower biomass yield during growth on gluconeogenic substrates (16–18). We show here that P. putida, in contrast, curbed protein investment during growth on a gluconeogenic substrate, indicating a metabolic conditioning to maximize growth when fed on either glycolytic or gluconeogenic carbon substrates.

Evolutionarily, the oxidative branch of the PP pathway is considered a newer metabolic strategy due to its absence in a number of thermophilic organisms, archaea, and aerobic bacteria (52). Among proteobacterial species, *Sphingobium* spp. and Acinetobacter spp. also lack both G6P dehydrogenase and 6-phosphogluconolactonase for the initial reaction steps of the oxidative PP pathway, similar to *Comamonas* spp. (Fig. 1a and Table S1). Other species, such as *Cupriavidus* spp., *Zymomonas* spp., *Rhodobacter* spp., and *Delftia* spp., lack the 6-phosphogluconate dehydrogenase required for the last step of the oxidative PP pathway toward the production of ribose-5-phosphate (Fig. 1a and Table S1). In the aforementioned species, the decoupling of the EMP and ED pathways in concert with the absence of a complete oxidative PP pathway has been found to be paired with physiological preference for gluconeogenic substrates, which was evident from higher growth rates and greater biomass production relative to glycolytic substrates (Fig. 1d) (17, 53, 54). Our findings further revealed that remodeling metabolism to decouple the ED and EMP pathways in P. putida achieved a similar fast-growth phenotype during gluconeogenic growth relative to glycolytic growth (Fig. 1d). In sum, our multiomics investigation of cellular metabolism across two *Proteobacteria* with distinct metabolic networks provides a new systems-level mechanistic understanding of the partitioning of gluconeogenic fluxes between biosynthetic demand and energy production to promote a fast-growth phenotype. Therefore, the present findings will be instrumental in subsequent systems-level investigations of the metabolic network reprogramming in proteobacterial species with gluconeogenic carbon preference, especially those with biotechnological relevance.

## MATERIALS AND METHODS

### Phylogenetic analysis.

Species in the phylum *Proteobacteria* were selected to account for biotechnologically important species spanning *Alphaproteobacteria*, *Betaproteobacteria*, and *Gammaproteobacteria*. Additionally, three to four species within the same genera as P. putida KT2440 and *C. testosteroni* KF-1 were selected to illustrate physiological consistency within the genera. Genomes of selected species were retrieved from GenBank and analyzed on KBase to create a SpeciesTree using FastTree 2 (55, 56). Branch support values were estimated using 1,000 iterations (57). Multiple sequence alignment was conducted across 49 core genes defined by Clusters of Orthologous Groups (55). The presence or absence of physiological traits (glycolytic EMP, oxidative PP pathway, ED pathway, carboxylate transporters, and carbohydrate transport systems) was examined from the genome using the Kyoto Encyclopedia of Genes and Genomes (KEGG) (57–59) and MetaCyc (60) (Table S1). The confirmation of glucose utilization was determined from examples in the literature (Table S1).

### Growth conditions.

Cells of *C. testosteroni* KF-1 (DSMZ 14576) and *C. testosteroni* T-2 (DSMZ 6577) were obtained from Deutsche Sammlung für Mikroorganismen und Zellkulturen (Braunschweig, Germany), and cells of P. putida KT2440 were obtained from American Type Culture Collection (Manassas, VA). Cells were stored at −80°C in nutrient-rich broth and 25% glycerol between experiments. Batch growth experiments of the bacterial strains were conducted in triplicate in an incubator (model I24; New Brunswick Scientific, Edison, NJ) set at 30°C with shaking (220 rpm). Cells were grown on pH-adjusted (7.0) and filter-sterilized (Waters Corporation; 0.22-μm nylon filters) minimal-nutrient medium that contained the carbon substrate at 100 mM C (succinate or gluconate). The minimal-nutrient medium consisted of 5.0 mM NaH_2_PO_4_, 20 mM K_2_HPO_4_, 37 mM NH_4_Cl, 17 mM NaCl, 0.81 mM MgSO_4_ · 7H_2_O, and 34 μM CaCl_2_ · 2 H_2_O as well as essential trace metal nutrients as previously reported (61). Aliquots of frozen stocks were first transferred to minimal-nutrient medium in tubes and grown to the late exponential phase before being transferred into 125-mL or 250-mL baffled flasks at one-fifth volume for experimental conditions. Additional washing of cells with minimal-nutrient medium to remove extracellular matrix was conducted between transfers for specified experiments. Cell growth was monitored on an Agilent Cary UV-visible spectrophotometer (Santa Clara, CA) at an optical density (OD) of 600 nm. Growth rates were determined by fitting the time points taken during the exponential growth phase to the balanced growth model described previously (Fig. S1) (62). The conversion factor between OD and cell dry weight in grams (g_CDW_) was determined by lyophilizing cell pellets throughout growth as previously described (5). The linear fit between OD and g_CDW_ was determined with regression analysis (R^2^ coefficient greater than 0.750).

### Quantification of substrate consumption rates and metabolite secretion rates.

Aliquots of cell cultures were collected periodically throughout growth, filtered (Costar Spin-X 0.22-μm-pore-size filter), and stored at −20°C until liquid chromatography–high-resolution mass spectrometry (LC-HRMS) analysis. Concentrations of substrates and extracellular metabolites were determined from standards prepared from commercial chemicals (Millipore-Sigma, St. Louis, MO, or Fisher Scientific, Pittsburgh, PA). Extracellular samples were diluted to maintain concentrations within the standard range. Quantification of metabolite concentrations was conducted with a Thermo Scientific Xcalibur 3.0 Quan browser. Regression analysis on the substrate depletion and metabolite production over time was performed to determine consumption and secretion rates, respectively, normalized to biomass growth. Biomass yield (Y_X/S_) was determined from the linear fit of the substrate concentration and g_CDW_ determined from sample aliquots corresponding to the same time point throughout exponential growth.

### Isotope labeling and intracellular metabolite extraction.

During exponential growth, cells were filtered, lysed, and extracted as described previously (5). In brief, cells adhered to the filters were rapidly quenched with 2:2:1 cold (4°C) methanol:acetonitrile:water. The lysed cell particulates were pelleted via centrifugation of the liquid suspensions at 9,000 × *g* and 4°C. Aliquots of the supernatants were then dried under N_2_ gas before storage at −20°C until LC-HRMS analysis. Following analysis and identification of intracellular metabolites by LC-HRMS, metabolite pools and ^13^C-labeling fractions were extracted with the Metabolomic Analysis and Visualization Engine (MAVEN) software (63, 64). Quantification of intracellular pools was obtained using commercial standards (Millipore-Sigma or Fisher Scientific) with an R^2^ coefficient of 0.992 or higher for the calibration curve. Energy charge was calculated as described previously (36, 37) from the quantified pools of adenosine nucleotides using the following formula (equation 1):

(1) $$\frac{\left[\text{ATP}]+0.5[\text{ADP}\right]}{\left[\text{ATP}]+[\text{ADP}]+[\text{AMP}\right]}$$

For ^13^C labeling experiments, the unlabeled succinate in the minimal-nutrient medium was replaced with 100 mM C of [U-^13^C_4_]-succinate, [1,4-^13^C_4_]-succinate, or [2,3-^13^C_4_]-succinate obtained from Cambridge Isotopes (Tewksbury, MA). To confirm pseudo-steady-state labeling, cells were harvested at two OD at 600 nm (OD_600_) values, ∼0.5 and ∼1.0 (Fig. S5). The natural abundance of ^13^C in all isotopologue data was corrected using IsoCor v2 (65). To evaluate potential carry-over and subsequent incorporation of unlabeled carbons from the stock solution medium (containing LB-glycerol, less than 1% vol/vol under experimental conditions), *C. testosteroni* KF-1 cells were washed with minimal-nutrient medium between transfers onto [U-^13-^C_4_]-succinate. An additional experiment was conducted with *C. testosteroni* KF-1 cells from the LB-glycerol stock by pelleting and washing cells before a comparable concentration of LB-glycerol was re-added to the first transfer on [U-^13-^C_4_]-succinate (Fig. S4).

10.1128/mbio.03259-21.7 FIG S4 Direct transfers of *C. testosteroni* KF-1 cells compared to when cells were washed with minimal-nutrient medium between both transfers or a single transfer. (A) Schematics of experimental setups with transfers into [U-^13^C]-succinate and (B) the corresponding experimental isotopologue data for selected metabolites in the TCA cycle and EMP pathway. Error bars represent the standard deviation of the mean of three biological replicates. Download FIG S4, TIF file, 0.8 MB. Copyright © 2021 Wilkes et al. 2021 Wilkes et al. https://creativecommons.org/licenses/by/4.0/ This content is distributed under the terms of the Creative Commons Attribution 4.0 International license.

### Metabolomics analysis with LC-HRMS.

All intracellular and extracellular metabolite extracts were analyzed via an ultra-high-performance LC (Thermo Scientific DionexUltiMate 3000) coupled to a high-resolution MS (Thermo Scientific Q Exactive quadrupole-Oribitrap hybrid MS) with electrospray ionization as previously described (61). Briefly, reversed-phase ion-pairing chromatography was utilized at a flow rate of 0.180-mL min^−1^ with a C18 Acquity UPLC Waters (Milford, MA) column maintained at 25°C. For the MS, full-scan negative mode was employed. Accurate mass and standard retention time matches were used to identify metabolites.

### ^13^C-metabolic flux analysis.

Metabolic network models consisting of 63 reactions for P. putida KT2440 and 51 reactions for *C. testosteroni* KF-1 were constructed using gene annotations of metabolic enzymes reported in MetaCyc (60) and UniProt (66). Additionally, the stoichiometric ratios of metabolite precursors in central carbon metabolism to macromolecular biomass components were determined from the annotated genomes of each species. Due to the current lack of genome information for *C. testosteroni* T-2, the network model of *C. testosteroni* T-2 was assumed to be the same as that of *C. testosteroni* KF-1. The biomass efflux rates were calculated separately for each substrate and species using the experimentally obtained growth rates and previously reported macromolecular biomass composition (the mass ratio of RNA, DNA, protein, lipid, and polysaccharide to total biomass) (67–69). The biosynthetic demand from the TCA cycle, the PP pathway, and the EMP pathway were calculated by summing the total carbon biomass efflux rates of metabolites from each respective pathway. The anabolic demand for cofactor balance in each species was calculated using the growth rates, biomass stoichiometry, and reaction network (Data Set S1). The ATP cost for polysaccharide, protein, RNA, and DNA polymerization reactions was estimated using conversion factors (70).

Importantly, we note that the macromolecular biomass compositions have not yet been specifically characterized during growth on succinate or gluconate for all biomass components in either organism. For *C. testosteroni*, we used the phospholipids and fatty acids characterized in *C. testosteroni* P15 grown on succinate (68) and the composition determined for *C. testosteroni* ACM 4769 for the remainder of the cellular components (69). For P. putida KT2440, we used the comprehensive analysis of biomass composition obtained during growth on glucose (67). As demonstrated previously (71), we assumed that the biomass growth rate rather than substrate condition was the determining factor in the biosynthetic demand.

The software OpenFlux2 (72) was used to quantitate metabolic fluxes in central carbon metabolism. OpenFlux2 extends the elementary metabolic unit algorithm to create model-simulated isotopomer balances across parallel-labeling experiments (72). We optimized the ^13^C metabolic flux analysis (^13^C-MFA) across two labeling schemes ([1,4-^13^C_4_]-succinate and [2,3-^13^C_4_]-succinate) and three replicates per labeling scheme (six sets of isotopomer data in total), using the ^13^C-labeling patterns of metabolites (G6P, F6P, Xu5P, R5P, and S7P, malate fumarate, aspartate, citrate, αKG, phosphoenolpyruvate, 3-phosphoglycerate, dihydroxyacetone-3-phosphate) directly in the TCA cycle, EMP pathway, and PP pathway. The species-specific models were fitted to the measured mass isotopomer distributions, the quantified secretion rates, and the calculated biosynthetic efflux for each species. For the biosynthetic efflux, we allowed an error imprecision of two standard deviations to account for possible differences in the biomass efflux for the cells grown on succinate instead of glucose. Production and consumption rates of cofactors (NADH/UQH_2_ and NADPH) and ATP were calculated from the optimized fluxes of relevant reactions (Data Set S1). Potential influxes of unlabeled substrates from the extracellular media were left unbounded in the model if the nonlabelled fraction in the closest corresponding metabolite was greater than 0.01. For instance, we found low fractions of unlabeled malate (5%), pyruvate (5%), G6P (2%), DHAP (3%), and acetyl-CoA (2%) in P. putida KT2440 and thus included an input reaction to these metabolites in the model. Flux estimation was computed using 100 iterations starting from random initial values for all fluxes to find a global solution. Optimization of the metabolic flux analysis was evaluated by assessing the variance-weighted sum of squared residuals between the experimentally determined and model-estimated efflux rates and isotopomer distributions. In all cases, a statistically acceptable fit was obtained when the minimized sum of squared residual values was below the χ^2-^statistical test cutoff at the 95% confidence level. The optimized metabolic fluxes and their corresponding 95% confidence intervals determined from a nonlinear search algorithm are available in Data Set S1. The standard deviation (or individual flux precision) was calculated as previously reported (73). A flux precision scoring metric (*P*) between the fluxes determined for the conditions of washed and unwashed *C. testosteroni* KF-1 cells was calculated using the following formula (74) (equation 2):

(2) $$P={\left(\frac{{\left(UB_{95}-LB_{95}\right)}_{\text{unwashed}}}{{\left(UB_{95}-LB_{95}\right)}_{\text{washed}}}\right)}^{2}$$

### Proteomics analysis.

Cells (15 mL) of P. putida KT2440 and *C. testosteroni* KF-1 were collected in quadruplicate replicates during exponential growth (OD_600_ of about 1.0) on media containing 100 mM C of succinate or gluconate. After centrifugation and removal of the supernatant, cell pellets were stored at −80°C until extraction. The cell pellets were extracted by vortexing and heating (95°C, 20 min) in a reducing and denaturing SDS (1%)/Tris (200 mM, pH 8.0)/dithiothreitol (DTT; 10 mM) buffer and cysteine thiols alkylated with 40 mM iodoacetamide. Proteins were purified using a modified eFASP (enhanced filter-aided sample preparation) protocol (75), using Sartorius Vivacon 500 concentrators (30-kDa nominal cutoff). Proteins were digested with MS-grade trypsin (37°C, overnight), and peptides were eluted from the concentrator dried by vacuum centrifugation. For quantitative analysis, peptides were isotopically labeled at both N- and C termini using the diDO-IPTL methodology (76). Briefly, C termini were labeled with either oxygen-16 or -18 by enzymatic exchange in isotopic water of >98 atom% enrichment. N termini were labeled with either un- or dideuterated formaldehyde via reductive alkylation using sodium cyanoborohydride. Peptide extracts from each sample were split, and aliquots labeled were separately with CD_2_O/^16^O and CH_2_O/^18^O; the latter were pooled to serve as a common internal standard for quantification. Aliquots of the ^16^O-labeled peptides and ^18^O-labeled internal standard were mixed 1:1 vol/vol and analyzed by LC-MS for protein expression quantification.

For LC-MS analysis, peptide samples were separated on a monolithic capillary C_18_ column (GL Sciences Monocap Ultra, 100-μm inside diameter [i.d.] × 200-cm length) using a water-acetonitrile + 0.1% formic acid gradient (2% to 50% AcN over 180 min) at 360 nL/min using a Dionex Ultimate 3000 LC system with nanoelectrospray ionization (Proxeon Nanospray Flex source). Mass spectra were collected on an Orbitrap Elite mass spectrometer (Thermo Fisher) operating in a data-dependent acquisition (DDA) mode, with one high-resolution (120,000 *m*/Δ*m*) MS1 parent ion full scan triggering 15 rapid-mode tandem mass spectra (MS2) collision-induced dissociation (CID) fragment ion scans of selected precursors. Proteomic mass spectral data were analyzed using MorpheusFromAnotherPlace (MFAP) (76), using the predicted proteome of P. putida KT2440 or *C. testosteroni* KF-1 as a search database. Precursor and product ion mass tolerances for MFAP searches were set to 20 ppm and 0.6 Da, respectively. Static cysteine carbamidomethylation and variable methionine oxidation, N-terminal (d4)-dimethylation, and C-terminal ^18^O_2_ were included as modifications. The false-discovery rate for peptide-spectrum matches was controlled by target-decoy searching to <0.5%. Protein-level relative abundances and standard errors were calculated in R using the Arm postprocessing scripts for diDO-IPTL data (76; github.com/waldbauerlab).

### Statistical analysis.

We used one-way analysis of variance (ANOVA) combined with Tukey’s honestly significant difference (HSD) *post hoc* tests to determine statistically significant differences in the growth phenotypes and metabolite pools across substrates and species. A *P* value less than 0.05 was considered a statistically significant difference. Significantly differential protein expression between experimental conditions was determined by calculating a *Z*-score for protein abundance differences by taking the difference in the mean (log_2_-transformed) protein abundance between conditions and dividing it by the sum of the estimated total uncertainly for that protein in the two conditions. This estimated total uncertainty for a given condition was taken as the root-square sum of (i) the standard deviation of a protein’s abundance across the biological replicates of that condition plus (ii) the average standard error of the protein’s abundance across quantified spectra within each replicate. These *Z*-scores were converted to *P* values assuming a standard normal distribution, and then the familywise error rate for significantly differential expression between conditions was controlled to 0.05 using the *q* value method to correct for multiple testing (77).

### Data availability.

Proteomic mass spectral data are available via ProteomeXchange under accession number PXD027036 and the MassIVE repository (massive.ucsd.edu) under accession number MSV000087734. Metabolomics LC-HRMS data are available in the MetaboLights repository (www.ebi.ac.uk/metabolights/MTBLS3046) under the accession number MTBLS3046.
