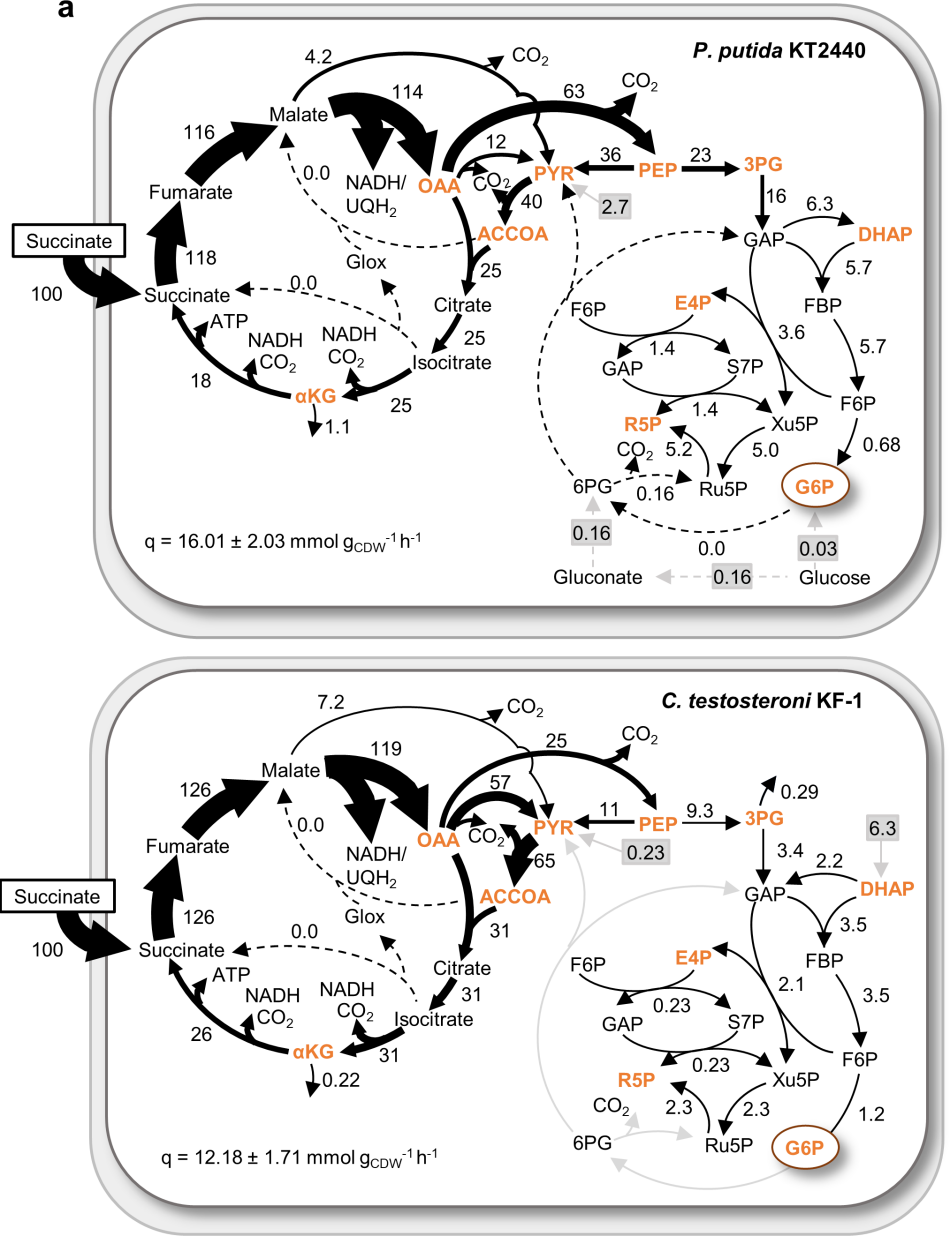# Erratum for Wilkes et al., “Analogous Metabolic Decoupling in *Pseudomonas putida* and *Comamonas testosteroni* Implies Energetic Bypass to Facilitate Gluconeogenic Growth”

**DOI:** 10.1128/mbio.03352-23

**Published:** 2024-02-05

**Authors:** Rebecca A. Wilkes, Jacob Waldbauer, Ludmilla Aristilde

## ERRATUM

Volume 12, no. 6, e03259-21, 2021, https://doi.org/10.1128/mbio.03259-21. Page 8, “Metabolic endpoint at G6P highlights…” paragraph 2, sentence 1: “false-[2,3-^13^C]-succinate” should read “[2,3-^13^C]-succinate.”

Page 9, Fig. 4a: The placements of citrate and isocitrate in the flux mapping were interchanged. Panel a should appear as shown below.