# Supplementary material for: Analogous Metabolic Decoupling in Pseudomonas putida and Comamonas testosteroni Implies Energetic Bypass to Facilitate Gluconeogenic Growth
Source: mBio. 2021 Dec 14;12(6):e03259-21. doi: 10.1128/mbio.03259-21 (PMC8669468; doi:10.1128/mbio.03259-21)
Supplement: TABLE S1 [file mbio.03259-21-st001.pdf]

Table S1. Functional traits determined for species shown in phylogenetic tree (see Fig. 1a in the main text). KEGG annotations for the genomes of each species were examined for the genes needed for a functional pathway.
*Used *C. thiooxydans* CNB-1 genome in KEGG database
**Used *C. testosteroni* TK102 for KEGG identifiers and MetaCyc to confirm for *C. testosteroni* KF-1

Species	Glycolytic EMP	Oxidative PP			ED pathway		C4-carboyxlate transporters	Carbohydrate transport systems				Glucose utilization
	PFK	G6PD	6PGL	6PGD	Edd	Eda	DctA	Glucose/ mannose	Ribose	Arabinose	Fructose PTS system	Example in literature
Zymomonas mobilis ZM4	NF	K00036	K01057	NF	K01690	K01625	NF	NF	NF	NF	NF	Positive (1)
Gluconobacter oxydans 621H	NF	K00036	K07404	K00033	K01690	K01625	NF	NF	K10439, K10440, K10441	NF	NF	Positive (2)
Sphingobium sp. SYK-6	K16370	K00036	NF	NF	K01690	K01625	K11103	NF	NF	NF	NF	Negative (3)
Rhodobacter sphaeroides ATCC 17029	K16370	K00036	K01057	NF	K01690	K01625	K11103	K10112	K10439, K10440, K10441	NF	Present	Positive (4)
Comamonas thiooxydans S23 *	K21071	NF	NF	NF	K01690	K01625	K11103	NF	NF	NF	NF	NF
Comamonas testosteroni KF-1 **	K21071	NF	NF	NF	K01690	K01625	K11103	NF	NF	NF	NF	Negative (this study)
Comamonas serinivorans SP-35	K21071	NF	NF	NF	NF	NF	NF	NF	NF	NF	NF	Negative (5)
Comamonas kerstersii J29	K21071	NF	NF	NF	NF	NF	K11103	NF	K10439, K10440, K10441	NF	NF	NF
Delftia acidovorans SPH-1	NF	K00036	K07404	NF	K01690	K01625	K11103	NF	K10439, K10440, K10441	NF	NF	Negative (6)
Cupriavidus necator H16 (*Ralstonia eutropha* H16)	NF	K00036	K01057	NF	K01690	K01625	K11103	NF	K10439, K10440, K10441	NF	NF	Negative (7)
Pseudomonas fluorescens F113	NF	K00036	K01057	K00033	K01690	K01625	K11103	K17315, K17316, K17317, K10112	K10439, K10440, K10441	K10537, K10538, K10539	K02770, K00882	Positive (8)
Pseudomonas alkylphenolica KL28	NF	K00036	K01057	K00033	K01690	K01625	K11103	K17315, K17316, K17317, K10112	K10439, K10440, K10441	NF	K02770, K00882	Positive (9)
Pseudomonas protegens Pf-5	NF	K00036	K01057	K00033	K01690	K01625	K11103	K17315, K17316, K17317, K10112	K10439, K10440, K10441	NF	K02770, K00882	Positive (10)
Pseudomonas putida KT2440	NF	K00036	K01057	K00033	K01690	K01625	K11103	K17315, K17316, K17317, K10112	K10439, K10440, K10441	NF	K02770, K00882	Positive (11)
Escherichia coli K12 MG1655	K16370	K00036	K07404	K00033	K01690	K01625	K11103	NF	K10439, K10440, K10441	K10537, K10538, K10539	K02770, K00882	Positive (12)
Acinetobacter baylyi ADP1	NF	NF	NF	NF	K01690	K01625	K11103	NF	NF	NF	K02770, K00882	Positive (13)

References

1. T. B. Jacobson, *et al.*, ^{2}H and ^{13}C metabolic flux analysis elucidates in vivo thermodynamics of the ED pathway in *Zymomonas mobilis*. *Metab. Eng.* **54**, 301–316 (2019).
2. T. Hanke, *et al.*, Combined fluxomics and transcriptomics analysis of glucose catabolism via a partially cyclic pentose phosphate pathway in *Gluconobacter oxydans* 621H. *Appl. Environ. Microbiol.* **79**, 2336–2348 (2013).
3. M. Varman, *et al.*, Decoding how a soil bacterium extracts building blocks and metabolic energy from ligninolysis provides road map for lignin valorization. *Proc. Natl. Acad. Sci.* **40**, E5802–E5811 (2016).
4. E. Orsi, *et al.*, Metabolic flux ratio analysis by parallel ^{13}C labeling of isoprenoid biosynthesis in *Rhodobacter sphaeroides*. *Metab. Eng.* **57**, 228–238 (2020).
5. D. Zhu, C. Xie, Y. Huang, J. Sun, W. Zhang, Description of *Comamonas serinivorans* sp. nov., isolated from wheat straw compost. *Int. J. Syst. Evol. Microbiol.* **64**, 4141–4146 (2014).
6. R. Shetty, *et al.*, Complete genome sequence of the phenanthrene-degrading soil bacterium Delftia acidovorans Cs1-4. *Stand. Genomic Sci.* **10**, 55 (2015).
7. S. Sichwart, S. Hetzler, D. Broker, A. Steinbuchel, Extension of the Substrate Utilization Range of *Ralstonia eutropha* Strain H16 by Metabolic Engineering To Include Mannose and Glucose. *Appl. Environ. Microbiol.* **77**, 1325–1334 (2011).
8. O. Rice, S. H. Miller, J. P. Morrissey, F. O'Gara, Exploitation of glucose catabolic gene fusions to investigate in situ expression during *Pseudomonas*-plant interactions. *Biol. Fertil. Soils* **48**, 235–238 (2012).
9. M. Mulet, *et al.*, *Pseudomonas alkylphenolica* sp. nov., a bacterial species able to form special aerial structures when grown on p-cresol. *Int. J. Syst. Evol. Microbiol.* **65**, 4013–4018 (2015).
10. R. A. Wilkes, C. M. Mendonca, L. Aristilde, A cyclic metabolic network in *Pseudomonas protegens* Pf-5 prioritizes the Entner-Doudoroff pathway and exhibits substrate hierarchy during carbohydrate coutilization. *Appl. Environ. Microbiol.* **85**, e02084-18 (2019).
11. P. I. Nikel, *et al.*, *Pseudomonas putida* KT2440 metabolizes glucose through a cycle formed by enzymes of the Entner-Doudoroff, Embden-Meyerhof-Parnas, and Pentose Phosphate pathways. *J. Biol. Chem.* **290**, 1–29 (2015).
12. W. D. Hollinshead, *et al.*, Examining Escherichia coli glycolytic pathways, catabolite repression, and metabolite channeling using Δpfk mutants. *Biotechnol. Biofuels* **9**, 212 (2016).
13. K. Salcedo-Vite, J.-C. Sigala, D. Segura, G. Gosset, A. Martinez, *Acinetobacter baylyi* ADP1 growth performance and lipid accumulation on different
