# Supplementary material for: Analogous Metabolic Decoupling in Pseudomonas putida and Comamonas testosteroni Implies Energetic Bypass to Facilitate Gluconeogenic Growth
Source: mBio. 2021 Dec 14;12(6):e03259-21. doi: 10.1128/mbio.03259-21 (PMC8669468; doi:10.1128/mbio.03259-21)
Supplement: TABLE S2 [file mbio.03259-21-st002.pdf]

Table S2A. Fold changes in protein abundances in succinate-grown relative to gluconate-grown *P. putida* KT2440. Values in bold represent *p* values considered significant after adjusting for the false discovery rate (q = 0.05). Data were obtained from four biological replicates. Not found, NF; Not applicable, N/A.

Reactions	ORF number	Protein name	Protein abundance $Log_2(FC)$	p value
Succinate_EX -> Succinate	PP_1188	Succinate transporter (DctA)	Succinate only	N/A
**Gluconate_EX -> gluconate**	**PP_3417**	**Gluconate transporter (GntT)**	**-3.140**	**0.0101**
Gluconate -> 6-phosphogluconate	PP_3416	Gluconokinase (GnuK)	Gluconate only	N/A
Gluconate -> 2-Ketogluconate	PP_3383	Gluconate 2-dehydrogenase (Gad)	Gluconate only	N/A
Gluconate -> 2-Ketogluconate	PP_3384	Gluconate 2-dehydrogenase (Gad)	NF	N/A
Gluconate -> 2-Ketogluconate	PP_3382	Gluconate 2-dehydrogenase (Gad)	NF	N/A
2-ketogluconate_peri -> 2-ketogluconate	PP_3377	2-ketogluconate transporter, putative (KguT)	Gluconate only	N/A
2-Ketogluconate -> 2-ketogluconate-6-phosphate	PP_3378	2-Ketogluconate kinase (KguK)	Gluconate only	N/A
2-ketogluconate-6-phosphate -> 6-phosphogluconate	PP_3376	2-Ketogluconate-6-phosphate reductase (KguD)	NF	N/A
Pyruvate ->Acetyl-CoA + CO_2	PP_0339	Pyruvate dehydrogenase (AceE)	0.304	0.339
Acetyl-CoA + Oxaloacetate -> Citrate + CoA	PP_2335	Citrate synthase (PrpC)	-0.343	0.334
acetyl-CoA + oxaloacetate -> Citrate + CoA	PP_4194	Citrate synthase (GltA)	0.00	0.399
citrate -> isocitrate	PP_2339	Aconitate hydratase B (aconitase) (AcnB)	-0.099	0.392
isocitrate -> α-ketoglutarate + CO_2	PP_4011	Isocitrate dehydrogenase [NADP] (Icd)	NF	N/A
isocitrate -> α-ketoglutarate + CO_2	PP_4012	Isocitrate dehydrogenase [NADP] (Idh)	0.279	0.377
α-Ketoglutarate -> Succinyl-CoA + CO_2	PP_4189	2-oxoglutarate dehydrogenase (SucA)	0.251	0.331
α-Ketoglutarate -> Succinyl-CoA + CO_2	PP_4188	2-oxoglutarate dehydrogenase (SucB)	-0.058	0.396
Succinyl-CoA -> Succinate	PP_4186	Succinyl-coenzymeA synthase (SucC)	0.194	0.371
Succinyl-CoA -> Succinate	PP_4185	Succinyl-coenzymeA synthase (SucD)	-0.187	0.363
**Succinate <-> Fumarate**	**PP_4191**	**Succinate dehydrogenase (SdhA)**	**0.978**	**0.0005**
Succinate <-> Fumarate	PP_4190	Succinate dehydrogenase (SdhB)	0.953	0.235
Fumarate <-> malate	PP_0897	Fumarate hydratase (FumA)	0.285	0.328
Malate + NAD+ <-> NADH + Oxaloacetate	PP_0654	Malate dehydrogenase (Mdh)	NF	N/A
Malate + a quinone = a quinol + Oxaloacetate	PP_0751	malate:quinone oxidoreductase (Mqo)	-0.952	0.031
Isocitrate -> Succinate + Glyoxylate	PP_4116	Isocitrate lyase (AceA)	-1.661	0.271
Acetyl-CoA + Glyoxylate -> Malate	PP_0356	Malate synthase (GlcB)	-0.458	0.253
**Pyruvate + CO_2 -> OAA**	**PP_5346**	**Pyruvate carboxylase (PycB)**	**-2.973**	**4.30E-06**

Oxaloacetate -> CO_2 + Pyruvate	PP_1389	Oxaloacetate decarboxylase (Odx)	0.338	0.357
GTP + Oxaloacetate -> CO_2 + GDP + Phosphoenolpyruvate	NF	PEP carboxykinase (Pck)	NF	N/A
Phosphoenolpyruvate -> Oxaloacetate	PP_1505	PEP carboxylase (Ppc)	NF	N/A
Malate <-> Pyruvate + CO_2	PP_5085	Malic enzyme (MaeB)	0.045	0.398
Pyruvate -> Phosphoenolpyruvate	PP_2082	Phosphoenolpyruvate synthase (PpsA)	-0.390	0.304
Phosphoenolpyruvate -> Pyruvate	PP_1362	Pyruvate kinase (PykA)	-0.095	0.395
Phosphoenolpyruvate <-> 2-Phosphoglycerate	PP_1612	Enolase (Eno)	0.239	0.370
2-Phosphoglycerate <-> 3-Phosphoglycerate	PP_5056	2,3-bisphosphoglycerate-dependent phosphoglycerate mutase (GpmI)	0.430	0.317
3-phosphoglycerate <-> 1,3-Bisphosphoglycerate	PP_4963	Phosphoglycerate kinase (Pgk)	0.053	0.396
**1,3-Bisphosphoglycerate + NADH -> Glyceraldehyde 3-phosphate + NAD+**	**PP_1009**	**Glyceraldehyde-3-phosphate dehydrogenase (GapA)**	**-4.403**	**6.09E-31**
1,3-Bisphosphoglycerate + NADH -> Glyceraldehyde 3-phosphate + NAD+	PP_2149	Glyceraldehyde-3-phosphate dehydrogenase (GapB)	0.615	0.203
Glyceraldehyde 3-phosphate <-> Dihydroxyacetone phosphate	PP_4715	Triosephosphate isomerase (TpiA)	0.032	0.398
Fructose 1,6-bisphosphate <-> Glyceraldehyde 3-phosphate + Dihydroxyacetone phosphate	PP_4960	Fructose-1,6-bisphosphate aldolase (Fba)	0.155	0.386
Fructose 1,6-bisphosphate -> Fructose 6-phosphate	PP_5040	Fructose-1,6-bisphosphatase (Fbp)	0.591	0.331
Glucose 6-phosphate <-> Fructose 6-phosphate	PP_1808	Glucose-6-phosphate isomerase (Pgi1)	-0.157	0.394
Glucose 6-phosphate <-> Fructose 6-phosphate	PP_4701	Glucose-6-phosphate isomerase (Pgi2)	NF	N/A
**Glucose-6-phosphate -> 6-Phosphoglucono-1,5-lactone**	**PP_1022**	**Glucose-6-phosphate 1-dehydrogenase (ZwfA)**	**-3.607**	**2.78E-10**
**6-Phosphoglucono-1,5-lactone -> 6-Phosphogluconate**	**PP_1023**	**6-phosphogluconolactonase (Pgl)**	**-4.126**	**7.30E-20**
6-Phosphogluconate -> Ribulose 5-phosphate + CO_2	PP_4043	Putative 6-phosphogluconate dehydrogenase (GntZ)	-0.255	0.382
Ribulose 5-phosphate <-> Xylulose 5-phosphate	PP_0415	Ribulose-phosphate 3-epimerase (Rpe)	Succ only	0.399
Ribulose 5-phosphate <-> Ribose 5-phosphate	PP_5150	Ribose-5-phosphate isomerase A (Rpi)	0.321	0.353
Sedoheptulose 7-phosphate + Glyceraldehyde 3-phosphate <-> Ribose 5-phosphate + Xylulose 5-phosphate	PP_4965	transketolase (Tkt)	0.087	0.39
Erythrose 4-phosphate + Xylulose 5-phosphate <-> Fructose 6-phosphate + Glyceraldehyde 3-phosphate	PP_4966	transketolase (Tkt)	0.087	0.390
Glyceraldehyde 3-phosphate + Sedoheptulose 7-phosphate <-> Fructose 6-phosphate + Erythrose 4-phosphate	PP_2168	Transaldolase (Tal)	-0.801	0.158
6-phosphogluconate -> 2-Dehydro-3-deoxy-6-phosphogluconate	PP_1010	6-phosphogluconate dehydratase (Edd)	-2.949	**4.05E-09**
2-Dehydro-3-deoxy-6-phosphogluconate -> Glyceraldehyde 3-phosphate + Pyruvate	PP_1024	2-dehydro-3-deoxy-phosphogluconate aldolase (Eda)	-3.463	**2.37E-07**

Table S2B. Fold changes in protein abundances in succinate-grown relative to gluconate-grown *C. testosteroni* KF-1. Values in bold represent *p* values considered significant after adjusting for the false discovery rate (q = 0.05). Data were obtained from four biological replicates. Not found, NF; Not applicable, N/A.

Reactions	ORF number	Protein name	Protein abundance Log2(FC)	p-value
Succinate_EX -> Succinate	WP_042437594.1	Succinate transporter (DctA)	Succinate only	N/A
Gluconate_EX -> gluconate	WP_003060256.1	Gluconate transporter (GntT)	NF	N/A
Gluconate -> 6-phosphogluconate	WP_003053074.1	Gluconokinase (GnuK)	NF	N/A
Gluconate -> 2-Ketogluconate	WP_003052499.1	Gluconate 2-dehydrogenase (Gad)	NF	N/A
2-ketogluconate_peri -> 2-ketogluconate	WP_003073082.1	2-ketogluconate transporter, putative (KguT)	Gluconate only	N/A
2-Ketogluconate -> 2-ketogluconate-6-phosphate	NF	2-Ketogluconate kinase (KguK)	NF	N/A
2-ketogluconate-6-phosphate -> 6-phosphogluconate	NF	2-Ketogluconate -6-phosphate reductase (KguD)	NF	N/A
Pyruvate ->Acetyl-CoA + CO_2	WP_003079261.1	Pyruvate dehydrogenase (PDH)	0.021285179	0.398
acetyl-CoA + oxaloacetate -> Citrate + CoA	WP_003063579.1	Citrate synthase (GltA)	0.02	0.398
**citrate -> isocitrate**	**WP_003077707.1**	**Aconitate hydratase B (aconitase) (Acn)**	**-1.32148789**	**4.90E-04**
citrate -> isocitrate	WP_003074861.1	Aconitate hydratase B (aconitase) (AcnB)	0.742778393	0.016
isocitrate -> α-ketoglutarate + CO_2	WP_003075787.1	Isocitrate dehydrogenase [NADP] (IDH1)	-0.05327177	0.396
isocitrate -> α-ketoglutarate + CO_2	WP_003075779.1	Isocitrate dehydrogenase [NADP] (IDH2)	-0.47473197	0.153
α-Ketoglutarate -> Succinyl-CoA + CO_2	WP_003055529.1	2-oxoglutarate dehydrogenase (SucA)	-0.72855508	0.038
**α-Ketoglutarate -> Succinyl-CoA + CO_2**	**WP_003079542.1**	**2-oxoglutarate dehydrogenase (Suc)**	**-0.89460046**	**0.024**
Succinyl-CoA -> Succinate	WP_003060063.1	Succinyl-coenzymeA synthase (SucC)	0.792938396	0.004
Succinyl-CoA -> Succinate	WP_003060065.1	Succinyl-coenzymeA synthase (SucD)	0.585472077	0.176
Succinate <-> Fumarate	WP_003063573.1	Succinate dehydrogenase (SdhA)	0.624324295	0.218
Succinate <-> Fumarate	WP_042438405.1	Succinate dehydrogenase (SdhB)	0.540072365	0.193
Succinate <-> Fumarate	WP_003074853.1	Succinate dehydrogenase (SdhC)	0.028758807	0.399
Fumarate <-> malate	WP_003061052.1	Fumarate hydratase (FH1)	0.927807457	0.054
Fumarate <-> malate	WP_003078203.1	Fumarate hydratase (FH2)	-0.485187783	0.207
Malate + NAD+ <-> NADH + Oxaloacetate	WP_003053219.1	Malate dehydrogenase (Mdh)	0.272711414	0.308
Malate + a quinone = a quinol + Oxaloacetate	WP_003078637.1	malate:quinone oxidoreductase (Mqo)	Succinate only	N/A
Isocitrate -> Succinate + Glyoxylate	WP_003053566.1	Isocitrate lyase (AceA)	Gluconate only	N/A
Acetyl-CoA + Glyoxylate -> Malate	WP_003080923.1	Malate synthase (MS)	-0.20031641	.388
Pyruvate + CO_2 -> OAA		Pyruvate carboxylase (PycB)	NF	N/A

Oxaloacetate -> CO_2 + Pyruvate	WP_003054191.1	Oxaloacetate decarboxylase (Odx)	Gluconate only	N/A
**GTP + Oxaloacetate -> CO_2 + GDP + Phosphoenolpyruvate**	**WP_003072005.1**	**PEP carboxykinase (Pck)**	**4.72**	**1.59E-14**
Phosphoenolpyruvate -> Oxaloacetate	WP_003078754.1	PEP carboxylase (PEPcx)	Gluconate only	N/A
Malate <-> Pyruvate + CO_2	WP_003072131.1	Malic enzyme (ME1)	succ only	N/A
Malate <-> Pyruvate + CO_2	WP_003074720.1	Malic enzyme (ME2)	0.219	0.363
Pyruvate -> Phosphoenolpyruvate	WP_003076285.1	Phosphoenolpyruvate synthase (PEPs)	0.890	0.033
**Phosphoenolpyruvate -> Pyruvate**	**WP_034347964.1**	**Pyruvate kinase (PK)**	**-1.17**	**0.001**
Phosphoenolpyruvate <-> 2-Phosphoglycerate	WP_003057720.1	Enolase (Eno)	0.731	0.136
2-Phosphoglycerate <-> 3-Phosphoglycerate	WP_003072651.1	2,3-bisphosphoglycerate-dependent phosphoglycerate mutase (Gpm)	0.619	0.168
3-phosphoglycerate <-> 1,3-Bisphosphoglycerate	WP_003072267.1	Phosphoglycerate kinase (Pgk)	1.696	0.011
1,3-Bisphosphoglycerate + NADH -> Glyceraldehyde 3-phosphate + NAD+	WP_003060372.1	Glyceraldehyde-3-phosphate dehydrogenase (Gap)	0.376	0.279
Glyceraldehyde 3-phosphate <-> Dihydroxyacetone phosphate	WP_003057838.1	Triosephosphate isomerase (TpiA)	NF	N/A
Fructose 1,6-bisphosphate <-> Glyceraldehyde 3-phosphate + Dihydroxyacetone phosphate	WP_003072472.1	Fructose-1,6-bisphosphate aldolase (Fba)	0.479	0.136
Fructose 1,6-bisphosphate -> Fructose 6-phosphate	WP_003061245.1	Fructose-1,6-bisphosphatase (Fbp)	0.262	0.374
Glucose 6-phosphate <-> Fructose 6-phosphate	WP_029158506.1	Glucose-6-phosphate isomerase (Pgi1)	NF	N/A
6-Phosphogluconate -> Ribulose 5-phosphate + CO_2	WP_003050579.1	Putative 6-phosphogluconate dehydrogenase (GntZ)	NF	N/A
Ribulose 5-phosphate <-> Xylulose 5-phosphate	WP_003076181.1	Ribulose-phosphate 3-epimerase (Rpe)	NF	N/A
Ribulose 5-phosphate <-> Ribose 5-phosphate	WP_012838302.1	Ribose-5-phosphate isomerase A (RpiA)	0.349	0.349
Sedoheptulose 7-phosphate + Glyceraldehyde 3-phosphate <-> Ribose 5-phosphate + Xylulose 5-phosphate	WP_003072441.1	transketolase (Tkt)	0.081	0.396
Erythrose 4-phosphate + Xylulose 5-phosphate <-> Fructose 6-phosphate + Glyceraldehyde 3-phosphate	WP_003072441.1	transketolase (Tkt)	0.081	0.396
Glyceraldehyde 3-phosphate + Sedoheptulose 7-phosphate <-> Fructose 6-phosphate + Erythrose 4-phosphate	WP_003058705.1	Transaldolase (Tal)	NF	N/A
6-phosphogluconate -> 2-Dehydro-3-deoxy-6-phosphogluconate	WP_003074773.1	6-phosphogluconate dehydratase (Edd)	Gluconate only	N/A
2-Dehydro-3-deoxy-6-phosphogluconate -> Glyceraldehyde 3-phosphate + Pyruvate	WP_003074771.1	2-dehydro-3-deoxy-phosphogluconate aldolase (Eda)	Gluconate only	N/A
