# Supplementary material for: Analogous Metabolic Decoupling in Pseudomonas putida and Comamonas testosteroni Implies Energetic Bypass to Facilitate Gluconeogenic Growth
Source: mBio. 2021 Dec 14;12(6):e03259-21. doi: 10.1128/mbio.03259-21 (PMC8669468; doi:10.1128/mbio.03259-21)
Supplement: TABLE S3 [file mbio.03259-21-st003.pdf]

Table S3. Physiological characteristics of *P. putida* KT2440, *C. testosteroni* KF-1, and *C. testosteroni* T-2 grown on succinate or gluconate. NF indicates that the metabolites were not quantified in the extracellular medium. Data are expressed as mean ± the standard deviation of three biological replicates.
*Biomass yield accounted for 2-ketogluconate as a substrate in addition to gluconate.
**Data in parentheses represent consumption rate for 2-ketogluconate.

	P. putida KT2440	*C. testosteroni* KF-1	*C. testosteroni* T-2
**Succinate**			
Growth rate (h^{-1})	0.98 ± 0.05	0.67 ± 0.05	0.62 ± 0.04
Biomass yield (g_{CDW} $g_{Substrate}^{-1}$)	0.53 ± 0.07	0.40 ± 0.06	0.28 ± 0.08
Consumption rate (mmol g_{CDW}^{-1} h^{-1})	16.0 ± 2.0	12.2 ± 1.7	12.7 ± 1.3
Secretions (mmol g_{CDW}^{-1} h^{-1})			
α-Ketoglutarate	0.16 ± 0.04	0.027 ± 0.003	3.5 ± 2.4 x10^{-3}
Citrate	1.4 ± 0.5 x10^{-3}	NF	NF
Fumarate	0.48 ± 0.07	NF	NF
Malate	0.71 ± 0.35	NF	NF
Pyruvate	0.10 ± 0.04	NF	NF
Aspartate	0.015 ± 0.002	NF	NF
Glutamate	0.16 ± 0.03	5.6 ± 0.7 x10^{-3}	0.12 ± 0.05 x10^{-3}
**Gluconate**			
Growth rate (h^{-1})	0.64 ± 0.05	0.38 ± 0.01	0.53 ± 0.01
Biomass yield (g_{CDW} $g_{Substrate}^{-1}$)	0.23 ± 0.01*	0.14 ± 0.03	0.21 ± 0.02
Consumption rate (mmol g_{CDW}^{-1} h^{-1})	15.5 ± 1.8 (12.6 ± 2.6)**	10.47 ± 3.35	14.33 ± 3.97
Secretions (mmol g_{CDW}^{-1} h^{-1})			
2-Ketogluconate	8.8 ± 1.1	3.4 ± 0.4	6.3 ± 1.2
Succinate	5. 3 ± 3.9 x10^{-3}	1.7 ± 0.3 x10^{-3}	8.2 ± 1.0 x10^{-3}
3-Phosphoglycerate	0.012 ± 0.003	0.76 ± 0.50 x10^{-3}	1.1 ± 0.9 x10^{-3}
6-Phosphogluconate	1.7 ± 0.4 x10^{-3}	1.5 ± 0.4 x10^{-3}	1.8 ± 0.7 x10^{-3}
Pyruvate	NF	3.3 ± 0.5 x10^{-3}	2.6 ± 0.6 x10^{-3}
Glutamate	0.056 ± 0.024	4.2 ± 1.6 x10^{-3}	0.047 ± 0.008
Aspartate	NF	NF	4.9 ± 0.4 x10^{-3}
