# Supplementary figures and images for: Analogous Metabolic Decoupling in Pseudomonas putida and Comamonas testosteroni Implies Energetic Bypass to Facilitate Gluconeogenic Growth
Source: mBio. 2021 Dec 14;12(6):e03259-21. doi: 10.1128/mbio.03259-21 (PMC8669468; doi:10.1128/mbio.03259-21)

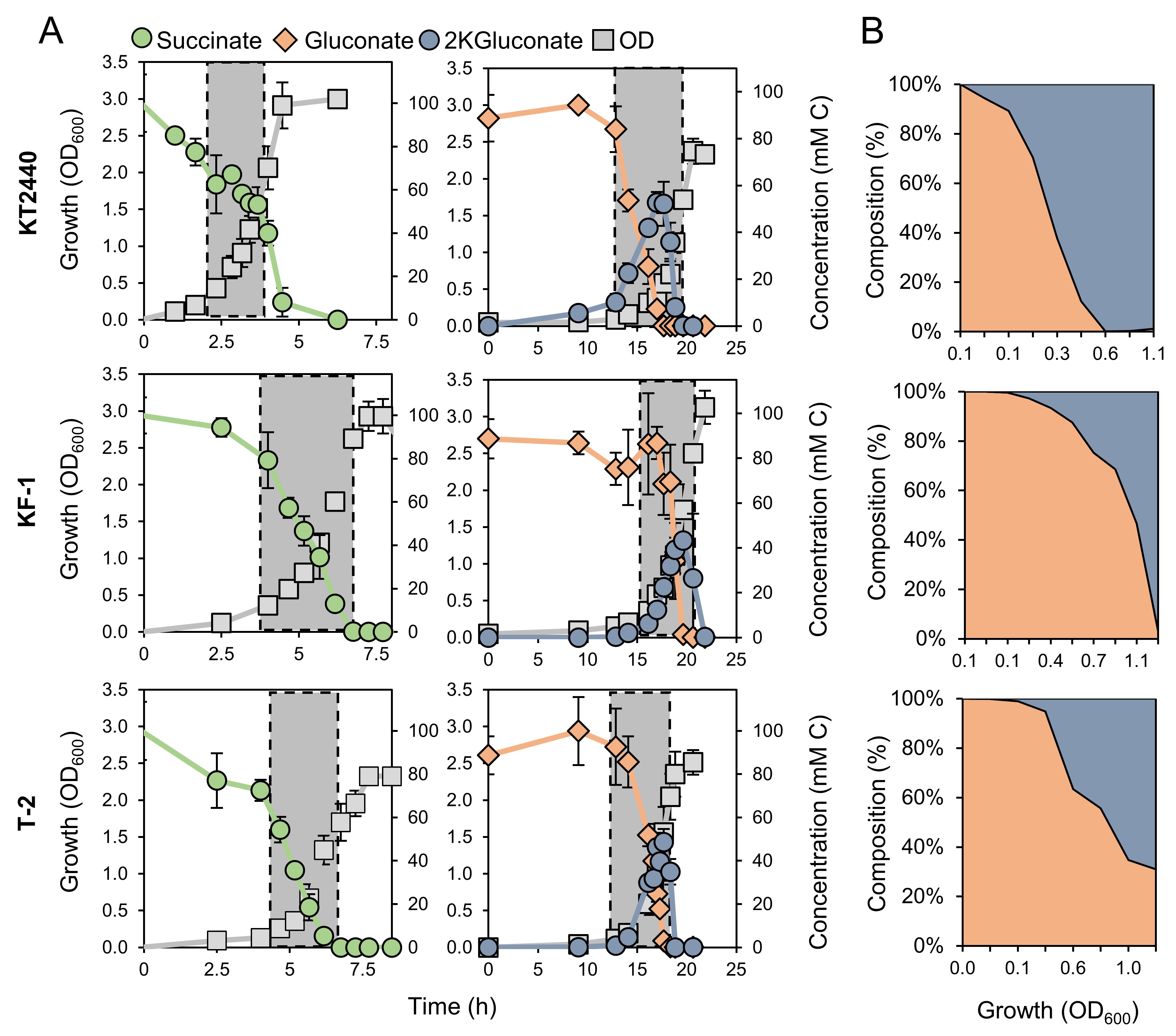

Supplement: FIG S1 [file mbio.03259-21-sf001.tif]

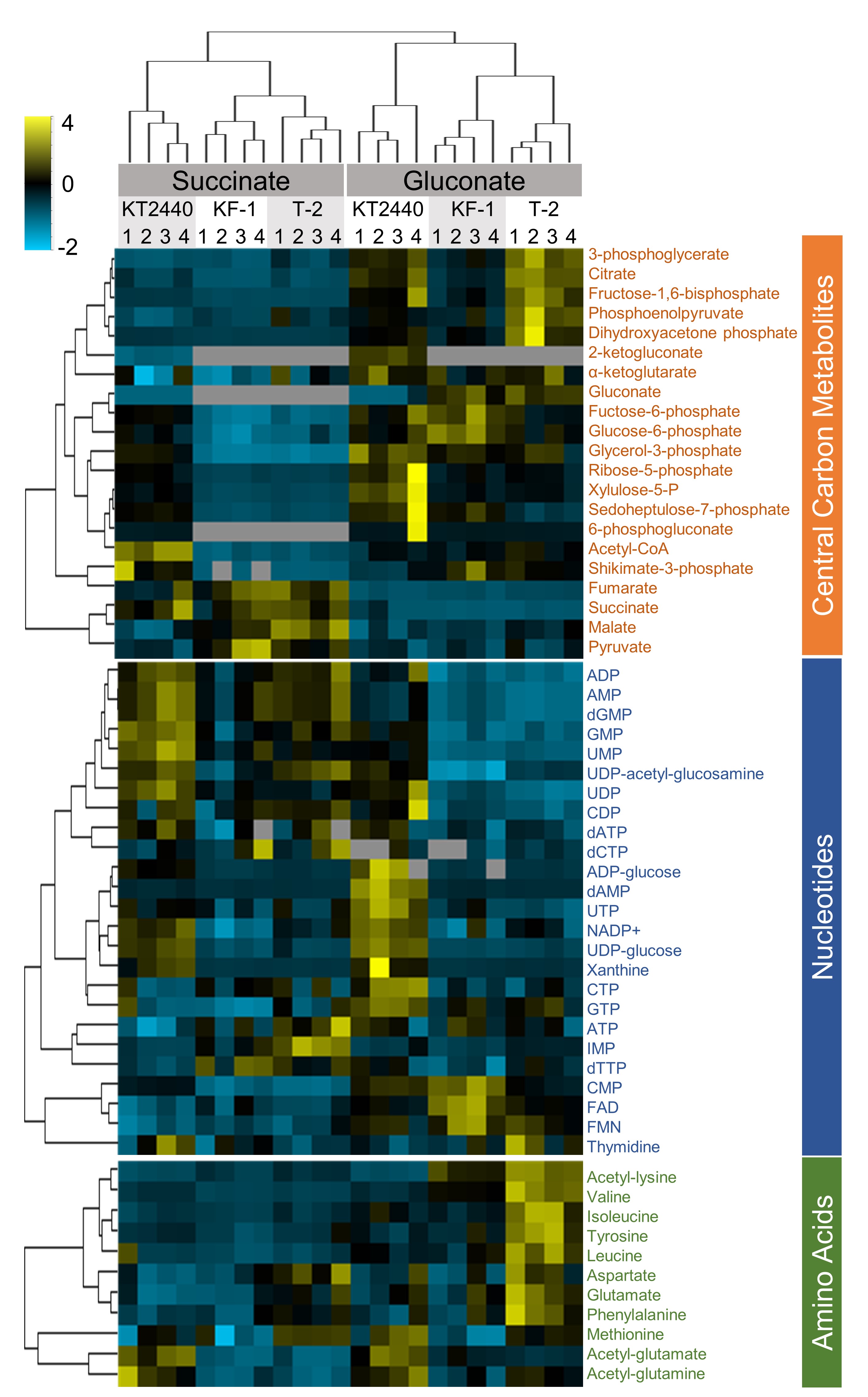

Supplement: FIG S2 [file mbio.03259-21-sf002.tif]

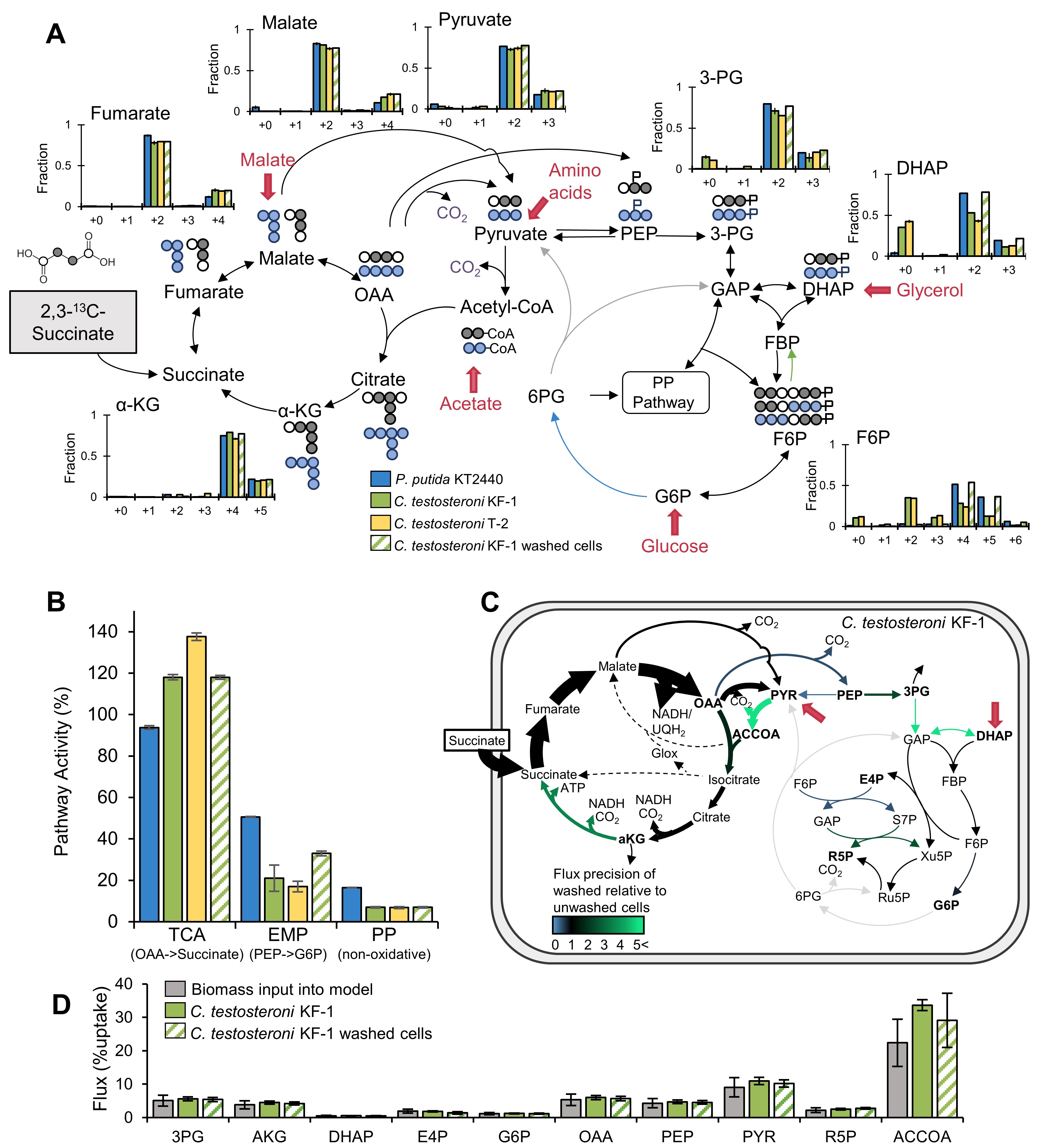

Supplement: FIG S3 [file mbio.03259-21-sf003.tif]

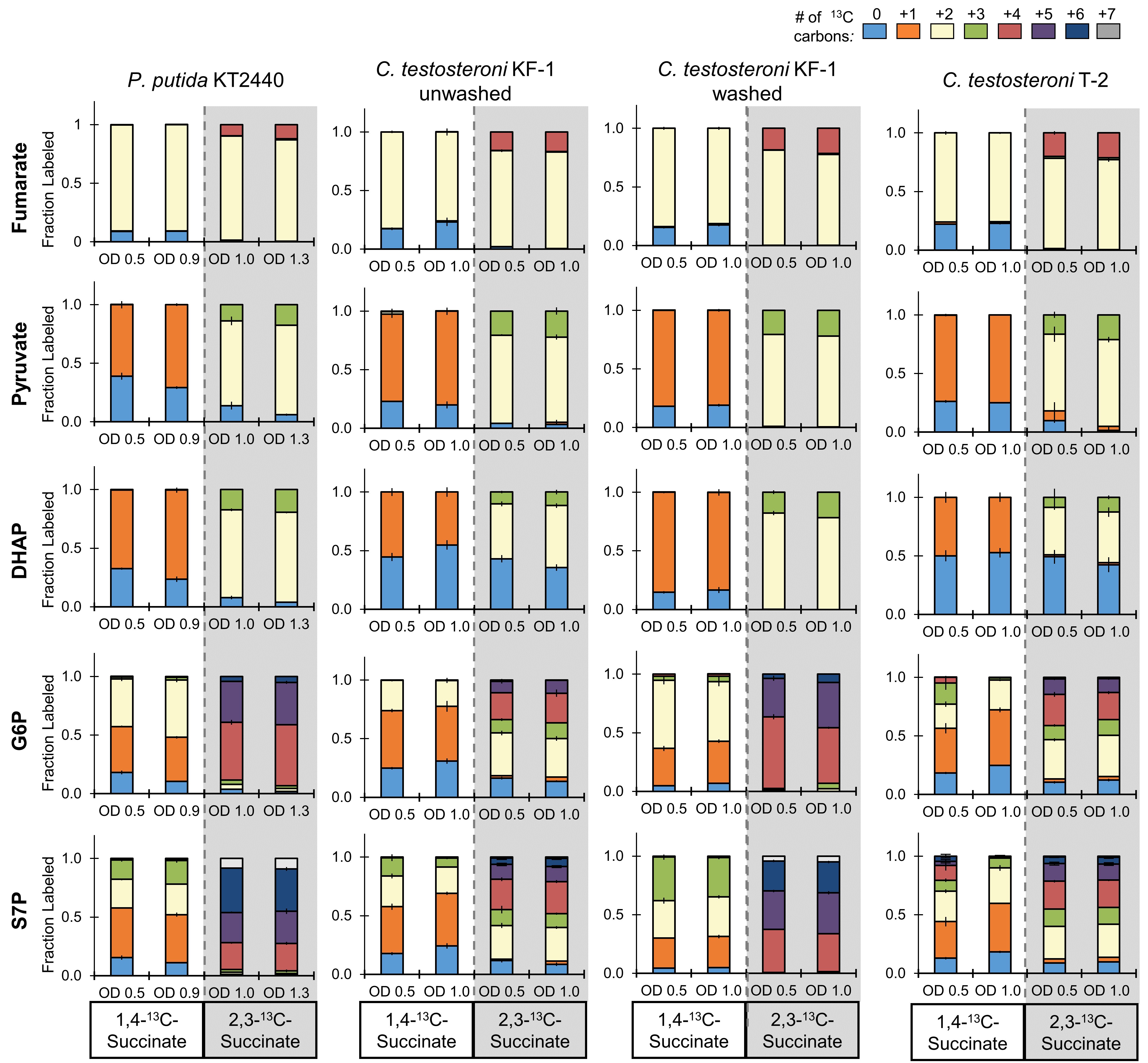

Supplement: FIG S5 [file mbio.03259-21-sf005.tif]

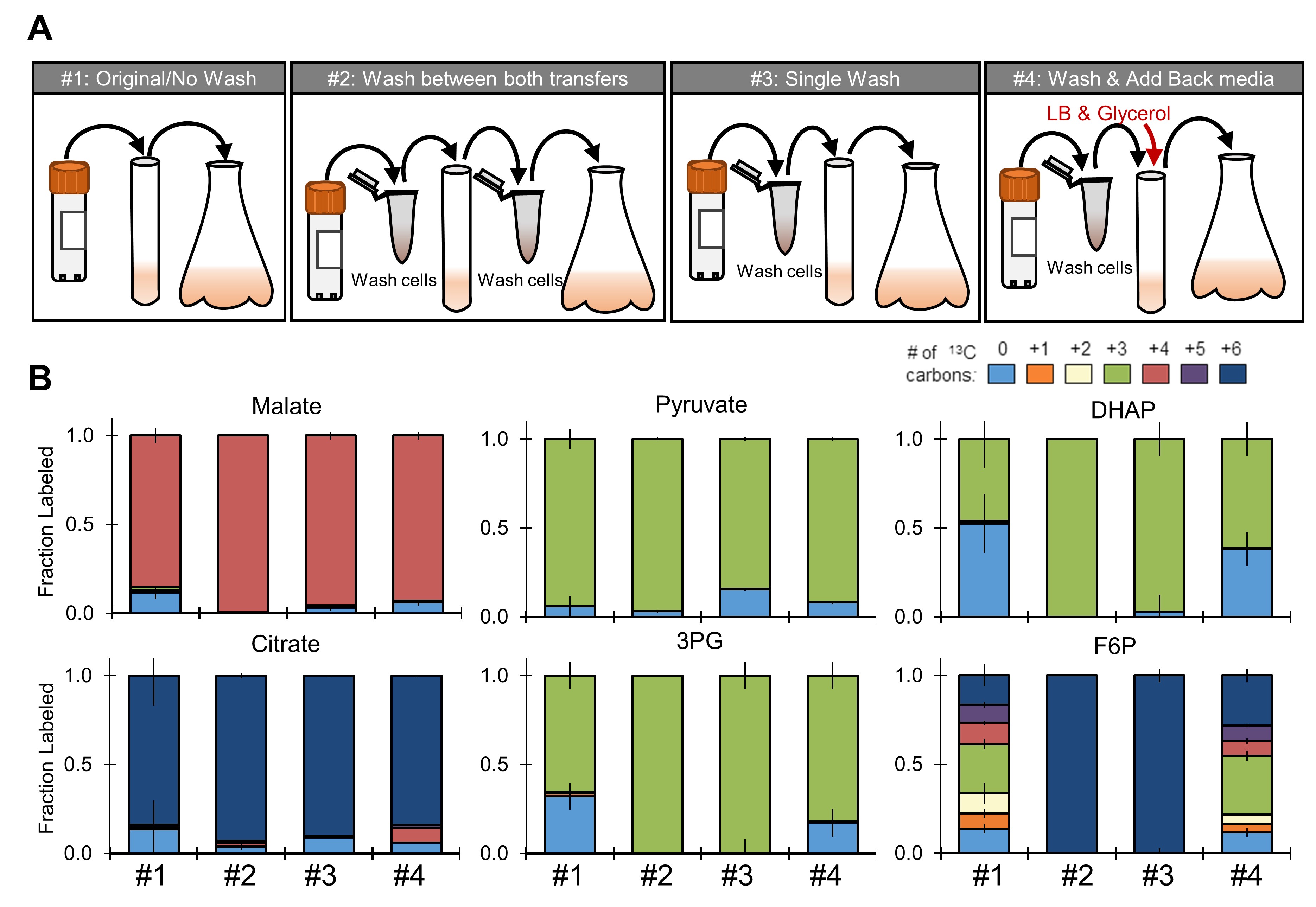

Supplement: FIG S4 [file mbio.03259-21-sf004.tif]
