# Supplementary material for: Analogous Metabolic Decoupling in Pseudomonas putida and Comamonas testosteroni Implies Energetic Bypass to Facilitate Gluconeogenic Growth
Source: mBio. 2021 Dec 14;12(6):e03259-21. doi: 10.1128/mbio.03259-21 (PMC8669468; doi:10.1128/mbio.03259-21)
Supplement: TEXT S1 [file mbio.03259-21-t0001.docx]

**Determination of carbon scavenging.** Carbon mapping and analysis of the isotopologue fractions in *C. testosteroni* KF-1 during growth on [2,3-^13^C]-succinate revealed large nonlabelled fractions up to 42% in dihydroxyacetone-3-phosphate (DHAP) and smaller fractions up to 15% in 3PG that could not be accounted for from carbon rearrangements (Fig. S3a and S5). Further, the nonlabelled fractions were consistent with the isotopologue fractions found for F6P and G6P, across multiple labeling schemes, and in the additional strain *C. testosteroni* T-2 (Fig. S3a and S5). Thus, we determined that the nonlabelled fractions were not instrumental artifact. We hypothesized that *C. testosteroni* scavenge for low abundance unlabeled extracellular carbons retained from the initial lysogeny broth (LB) and 25% glycerol stock, which was estimated to be less than 1% v/v in the experimental condition after two transfers of cells into the growth media. To test this hypothesis, we grew *C. testosteroni* KF-1 cells on [U-^13^C]-succinate and separately on [2,3-^13^C]-succinate with and without washing between transfers and found that the unlabeled fractions were not present when cells were washed (Fig. S3a, S4, and S5). To validate that the unlabeled fractions were from the initial LB glycerol stock, we washed cells before the first transfer into [U-^13^C]-succinate and added back in an equivalent concentration of LB and glycerol. We found that the unlabeled fractions returned supporting the hypothesis that the nonlabelled isotopologue fractions were from scavenged extracellular carbons in *C. testosteroni* (Fig. S4).

In comparison to when cells were washed between transfers, scavenging of peripheral carbons in *C. testosteroni* KF-1 supported a 10% greater total optimized biomass efflux (Fig. S3d). Modelling the isotope labeling of washed *C. testosteroni* KF-1 cells also indicated a 36% higher flux activity in the EMP pathway and a 16% lower biomass efflux to acetyl-CoA than the model of unwashed cells (Fig. S3b and S3d). Additionally, the flux model of *C. testosteroni* KF-1 when cells were washed between transfers had small improvements in the flux precision (Fig. S3c). Ultimately, we found that both *C. testosteroni* strains maintained a higher flux by as much as 47% in the TCA cycle and a lower flux by as much as 66% toward the EMP and PP pathway than *P. putida* (Fig. S3b).
